# Early and late feathering in turkey and chicken: same gene but different mutations

**DOI:** 10.1186/s12711-018-0380-3

**Published:** 2018-03-22

**Authors:** Martijn F. L. Derks, Juan M. Herrero-Medrano, Richard P. M. A. Crooijmans, Addie Vereijken, Julie A. Long, Hendrik-Jan Megens, Martien A. M. Groenen

**Affiliations:** 1 Wageningen University and Research Animal Breeding and Genomics, P.O. Box 338, 6700 AH Wageningen, The Netherlands; 2 Hendrix Genetics Turkeys, Technolgy and Service B.V., P.O. Box 114, 5830 AC Boxmeer, The Netherlands; 3 Animal Biosciences and Biotechnology Laboratory, Agricultural Research Service, US Department of Agriculture, Beltsville, MD 20705 USA

## Abstract

**Background:**

Sex-linked slow (SF) and fast (FF) feathering rates at hatch have been widely used in poultry breeding for autosexing at hatch. In chicken, the sex-linked *K* (SF) and *k*+ (FF) alleles are responsible for the feathering rate phenotype. Allele *K* is dominant and a partial duplication of the prolactin receptor gene has been identified as the causal mutation. Interestingly, some domesticated turkey lines exhibit similar slow- and fast-feathering phenotypes, but the underlying genetic components and causal mutation have never been investigated. In this study, our aim was to investigate the molecular basis of feathering rate at hatch in domestic turkey.

**Results:**

We performed a sequence-based case–control association study and detected a genomic region on chromosome Z, which is statistically associated with rate of feathering at hatch in turkey. We identified a 5-bp frameshift deletion in the *prolactin receptor* (*PRLR*) gene that is responsible for slow feathering at hatch. All female cases (SF turkeys) were hemizygous for this deletion, while 188 controls (FF turkeys) were hemizygous or homozygous for the reference allele. This frameshift mutation introduces a premature stop codon and six novel amino acids (AA), which results in a truncated PRLR protein that lacks 98 C-terminal AA.

**Conclusions:**

We present the causal mutation for feathering rate in turkey that causes a partial C-terminal loss of the prolactin receptor, and this truncated PRLR protein is strikingly similar to the protein encoded by the slow feathering *K* allele in chicken.

**Electronic supplementary material:**

The online version of this article (10.1186/s12711-018-0380-3) contains supplementary material, which is available to authorized users.

## Background

Sex identification is an important management factor within many commercial livestock operations. In poultry, sexing can be performed by examining feathering rate, a non-intrusive approach to separate males and females at hatch. In turkey and chicken layer breeds, sexing at hatch is crucial for production. However, for broiler breeds this method is mainly applied at the parent stock level. In chicken, the sex-linked dominant *K* locus, which is located on the Z-chromosome, is responsible for feather development and is associated with delayed emergence of primary and secondary flight feathers (SF), while the *k* + allele is associated with fast emergence (FF) of flight feathers [1]. The status at this locus is widely used for autosexing at hatch [1]. Elferink et al. [2] studied the molecular basis of the *K* allele and identified a 176-kb tandem duplication, which includes part of the genes *prolactin receptor* (*PRLR*) and *sperm flagellar 2* (*SPEF2*) that are associated with the *K* allele. Moreover, a molecular test was developed to distinguish between homozygous and heterozygous late feathering males [2]. The 176-kb duplication causes a 149-amino-acid (AA) C-terminal loss of the PRLR protein and is most likely the causal mutation for the SF phenotype [3]. PRLR is a receptor of the anterior pituitary hormone prolactin that belongs to the type I cytokine receptor family [4] and is involved in various physiological processes including many reproductive and developmental processes, such as hair/coat morphology [4]. The *PRLR* gene is widely expressed in all embryonic and somatic tissues and its expression is higher in SF than in FF chicks [5].

The domesticated turkey (*Meleagris gallopavo*), an important agricultural species and the second largest contributor to world poultry production [6], shows similar SF and FF phenotypes in some commercial lines [7], which are used for the same selection goal as in chicken, i.e. reliable and easy determination of sex at hatch. The SF phenotype differs between turkey and chicken with SF turkeys generally showing poor feathering even at a later age [7]. Zakrzewska et al. [7] suggested that the dominant sex-linked inhibited feathering (IF) allele *K* is responsible for the genetic feathering defect in turkey. Interestingly, expression of this defect ranges from almost complete absence of feathers to full feather covering at a later age (> 4 weeks of age), although until 4 weeks of age no apparent differences between SF birds were observed. Moreover, SF turkeys show inferior reproductive efficiency compared with FF turkeys [8] and differences in body weight and carcass characteristics [9]. The SF phenotype that is under study here differs from a late feathering phenotype that was described in turkey by Asmundson and Abbott [10], which consists in poor feathering at physical maturity (> 20 weeks of age). In chicken, the SF phenotype has been associated with the sex-linked allele *K*, whereas in turkey, the underlying genetic components and causal mutation have never been investigated. In this study, we used whole-genome sequence data that were obtained from either slow- or fast-feathering turkeys to perform a case–control genome-wide association study (GWAS) for feathering rate at hatch and to investigate its relation to the chicken allele *K*.

## Methods

### Dataset used for sequencing and mapping

We collected blood from 202 animals representing nine commercial turkey lines and that included 12 SF cases and 12 FF cases selected from the same line. For each sample, DNA was extracted and sequenced on the Illumina HiSeq 2000 sequencer, which generated paired-end 101 bp reads. We used the Sickle software to trim sequences [11], BWA-MEM (version 0.7.15) to map the whole-genome sequencing data to the turkey reference genome (Melgal5) [12], the Samtools dedup function to remove duplicate reads [13], the GATK IndelRealigner to perform local realignments of reads around indels [14] and Qualimap to obtain mapping statistics [15].

### Variant detection and post-processing

We performed population-based variant calling using the Freebayes software with the following settings: (1) min-base-quality 10 (to exclude alleles with support base quality < 10), (2) min-alternate-fraction 0.2 (at least 20% of the reads should support the alternate allele in order to evaluate the position), (3) haplotype-length 0 (to avoid generating haplotypes in VCF), (4) ploidy 2 (assuming diploid organism), and (5) min-alternate-count 2 (to have at least two reads that support the alternate allele in order to evaluate position) [16]. Post-processing was performed using bcftools [13], and variants that were located within 3 bp of an indel, or with a phred quality score and call rate lower than 20 and 0.7, respectively, were removed. The average call rate was about 0.985, and the average transition/transversion (TS/TV) ratio was 2.62, in line with previous findings in turkey [17].

### Population statistics

PCA analysis was performed using PLINK [18] on the filtered vcf files and plotted using the default R plotting utilities.

### Functional annotation of variants

SnpEffect [19] was used for variant annotation and the PROVEAN software for variant effect prediction in missense variants. The following variant classes were considered as potential candidate variants: missense, splice acceptor, splice donor, inframe indels, frameshift, stop lost, stop gained, and start lost variants.

### Association study and identification of candidate variants

Single locus associations on the genotypes called by freebayes were tested for SNPs and indels in PLINK using permutations to generate uncorrected and corrected p values [18]. p values were generated by applying the Fisher’s exact test and an adaptive Monte Carlo permutation test was performed with 5000 replications. Variants with a P lower than 1e-5 were considered significant. Manhattan plots were generated using qqman R package [20]. We selected all significant protein-altering variants and evaluated their putative effect on the protein based on PROVEAN scores and SnpEffect annotations. Moreover, gene ontology (GO) annotations were obtained from the Uniprot database [21]. Phenotype information on *PRLR* null-mutant mice was from Craven et al. [22]. The ClustalO alignment software [23] was used to align chicken and turkey *PRLR* sequences.

### CNV analysis

CNV-seq was used to perform CNV analysis using a log2-threshold of 0.6 and a *p* value threshold of 0.001 [24]. The optimum window size was automatically computed and ranged from 2.5 to 7.1 kb. The FF sample MG-WUR-121 and the SF sample MG-WUR-136 were used as control samples in CNV-seq analysis for analyses of SF and FF data, both exhibiting average to high coverage (see Additional file 1: Table S1). CNV-seq R utilities were used to plot the CNV events.

## Results

### Case–control sequencing and variant detection

To study the molecular mechanisms that underlie feathering rate at hatch in turkey, we selected 12 animals within each group (SF and FF) from one commercial line for whole-genome re-sequencing (WGS) (All female, [see Additional file 1: Table S1]). Moreover, DNA from 178 FF turkeys from various commercial turkey lines was sequenced for additional control samples. The SF turkeys in the population analysed here have phenotypes that are similar to those described for the dominant sex-linked IF allele *K* by Zakrzewska et al. [7]. Whole-genome DNA was sequenced and resulted in a total amount of 2.17 Tbp (tera base pairs) from 22.48 × 10^9^ paired-end 101 bp reads. Mapping was performed with BWA—mem (version 0.7.15) to the *Meleagris gallopavo* build 5 (Melgal5: [12]) reference genome with an average mappability and coverage of 98.38%, and 10.5×, respectively. We performed population-based variant calling using Freebayes [16]. Next, we filtered out variants with a low-quality (phred quality score < 20) or a call rate lower than 0.7, which resulted in 8,136,213 (post-filtering) variants including 6,595,059 SNPs, and 1,197,170 indels, with an average variant density of 8.4 variants per kb (see Additional file 1: Table S2). We performed PCA analysis on the 24 cases and control animals to assess population stratification; no distinct clustering was observed between the two groups (see Additional file 2: Figure S1).

### Functional annotation of variants

We used SnpEff to assign a range of functional classes to the identified variants [19]. The majority of the variants were located in intronic, ncRNA, or intergenic regions (see Additional file 1: Table S3). We identified 231,073 coding (90,370 protein-altering) variants with an overall missense/silent ratio of 0.545, which means that for every two silent mutations (synonymous) one missense mutation is found (see Additional file 1: Table S4).

### Genome-wide association study for feathering rate at hatch

**Fig. 1:**
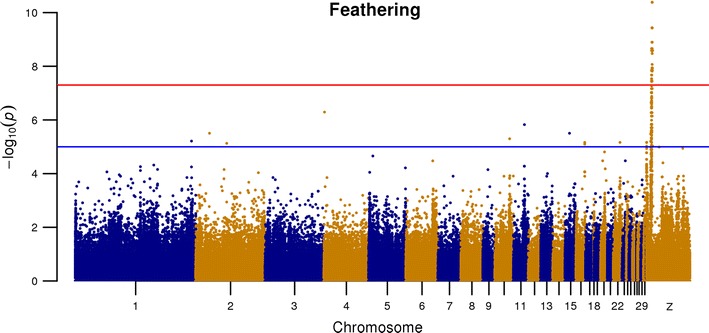
Manhattan plot for feathering rate association analysis. The − log10 (*P*) for each SNP is shown on the y-axis. A clear signal is observed on chromosome Z (8.1–9.5 Mb)

The GWAS revealed a significant signal for 134 SNPs on the Z chromosome. None of the detected variants is in perfect LD with the phenotype (see Additional file 1: Table S5). SNPs associated with the SF phenotype are all located on the short arm of the Z chromosome between 7.95 and 9.79 Mb (Fig. 1) and (see Additional file 2: Figure S2). This region contains 55 protein-coding genes including the *PRLR* and *SPEF2* genes associated with the SF phenotype in chicken.

### A 5-bp deletion in the *PRLR* gene is associated with slow feathering rate in turkey

**Fig. 2:**
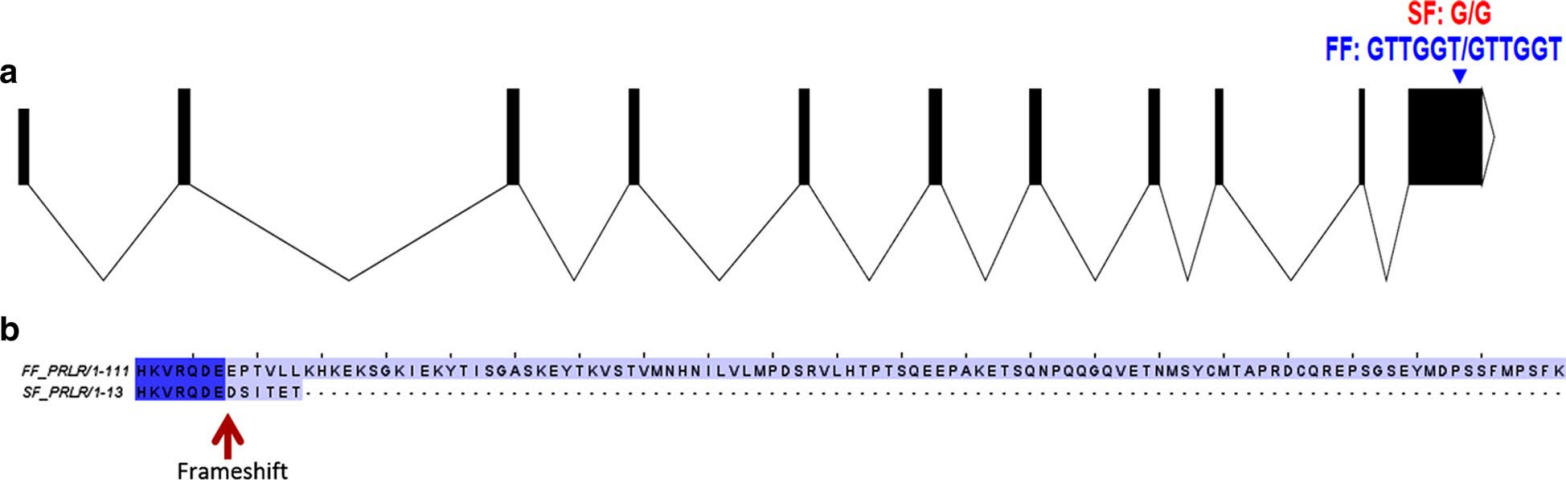
**a** *PRLR* gene model showing the location of the 5-bp deletion in the C-terminal exon. **b** C-terminal end of the PRLR protein sequence in SF and FF turkey. The deletion associated with SF phenotype introduces a frameshift and six novel AA before a premature stop-codon, which results in the deletion of 98 C-terminal AA

We examined the putative effects of all significant variants associated with slow feathering. In total, we identified eight protein-altering variants (seven SNPs and one indel). The seven identified SNPs cause missense mutations in protein coding genes (Table 1). None of the missense variants were predicted to have a high impact on the corresponding protein (reaching a PROVEAN score < − 2.5). Moreover, none of the missense mutations were fixed within the group of SF turkeys (Table 1), and thus were unlikely to be the causal variant. The identified indel represents a hemizygous 5-bp deletion that is statistically associated with feathering rate and predicted to have a high impact on the *PRLR* gene by causing a frameshift (Fig. 2). This deletion, which is located within the terminal exon of the *PRLR* gene, produces a truncated PRLR protein by introducing a premature stop codon and adding six novel C-terminal amino acids (DSITET*, Fig. 2). All SF turkeys were hemizygous for the alternate allele, while ten FF turkey controls and all additional 178 FF turkeys were hemizygous or homozygous for the reference allele (Table 2). In addition, we performed a copy-number variation (CNV) analysis to test whether, as in chicken, a CNV event is associated with feathering rate at hatch. Although one region on chromosome Z between 7.9 and 8.1 Mb harboured copy number variants in various samples, none of them were associated with feathering rate at hatch (see Additional file 3).

**Table 1:** Significant (p < 1e−5) protein-altering variants and predicted impact

Chr	bp	REF	ALT	P	Case/control AF	Gene	Type	Effect	Impact (PROVEAN)
**Z**	7,958,551	T	C	1.96e − 06	0.909/0.167	*RGP1*	Missense	Arg227Lys	Neutral (0.58)
**Z**	7,982,630	A	G	5.35e − 07	0.0833/0.833	*CREB3*	Missense	Pro178Ser	Neutral (− 1.55)
**Z**	7,982,834	A	G	1.96e − 06	0.909/0.167	*CREB3*	Missense	Val124Ile	Neutral (0.91)
**Z**	8,172,555	T	C	9.60e − 08	0.0833/0.917	*LOC104914814*	Missense	Gln47Arg	Neutral (− 0.22)
**Z**	8,181,148	A	G	5.35e − 07	0.917/0.167	*LOC100540309*	Missense	Arg157Lys	Neutral (0.90)
**Z**	8,227,879	T	C	5.35e − 07	0.0833/0.833	*LOC104914815*	Missense	Val320Ile	Neutral (− 0.07)
**Z**	9003502	A	T	2.60e − 09	0/0.833	*ADAMTS12*	Missense	Leu991Pro	Neutral (2.10)
**Z**	9426018	G	GTTGGT	2.60e − 09	1/0.167	*PRLR*	Frameshift	Glu704FS	High

**Table 2:** Genotypes of the 5-bp *PRLR* deletion for cases (SF) and control (FF) samples

Group	Phenotype	Number	Genotype		
			GTTGGT/GTTGGT or GTTGGT/-	GTTGGT/G	G/G or G/-
Cases	SF	12	0	0	12
Controls	FF	12	10	0	2
Test	FF	178	178	0	0

### Chicken and turkey slow feathering

Turkey and chicken PRLR proteins are very similar (90.24% sequence identity, (see Additional file 2: Figure S3) and both are 831 AA long. However, carriers of the 5-bp frameshift deletion in turkey lack the final 98 AA of the PRLR C-end tail (Fig. 2), whereas carriers of the *K* allele in chicken lack the terminal 149 AA of the PLRP C-end tail [3]. The prolactin receptor forms a dimer to bind prolactin in the extracellular space on the N-terminal end of the protein [4]. Moreover, PRLR contains two fibronectin type 3 domains (FN3), a WSXWS motif that is important for proper protein folding, and a Box 1 motif that is necessary for Janus kinase (JAK) interaction and activation [4]. However, the function of the affected C-end tail, which is located in the cytoplasm, is mostly unknown, but likely shares similar functional relevance in chicken and turkey.

## Discussion

This study reveals the molecular mechanisms that underlie the rate of feathering at hatch in turkey. The use of NGS data provided a sufficient number of variants to describe the potential causal polymorphism, i.e. a 5-bp deletion within the last exon of the *PRLR* gene. This mutation is different from that of allele *K* in chicken, but impacts the same gene [2] and moreover, in a similar manner, i.e. by loss of a substantial part of the C-end tail (98 AA in turkey; 149 AA in chicken). Unlike SF chickens, SF turkeys are poorly feathered, even at physical maturity [7]. Moreover, feathering of females can be so poor that carriers of this allele are not used commercially. Although strongly associated with SF, the *PRLR* 5-bp frameshift mutation is not in perfect LD with the phenotype since we observed two FF females that were hemizygous for this deletion. One possibility is that these two animals were mislabelled as FF turkeys, although they are SF turkeys; this is supported by the observation that none of the variants (including non-coding ones) is in complete LD with the phenotype.

The membrane-protein PRLR is a member of the cytokine receptor family that binds the prolactin hormone (PRL) within the extracellular space [4]. This hormone is involved in a diverse range of biological activities including various reproductive and developmental processes, such as hair replacement and follicle development [4]. Null mutant mice show different hair/coat morphologies and advanced hair replacement [25]. Moreover, a frameshift variant, which introduces a premature stop codon in the bovine PRLR receptor and causes the loss of 120 C-terminal AA, is associated with abnormally short and sleek hair coat [26]. Moreover, hair development and feather development are considered to have an evolutionary homologous origin. Thus, these findings support the *PRLR* gene as a likely candidate for feathering development within both commercial poultry species, chicken and turkey.

Other studies have suggested that feathering rate in chicken is caused by a higher expression of *PRLR* due to its partial duplication. Carriers of allele *K* show a 1.78-fold higher expression of *PRLR* in chicken [5]. In contrast, Zhao et al. [27] found no difference in *PRLR* expression between SF and FF chicks, but that the expression of the other gene involved in the duplication, *SPEF2*, was significantly higher in SF than in FF chicks, which suggested that a mutation in this gene was responsible for the SF phenotype. We believe that the higher expression of *SPEF2* in chicken is due to the large duplication that underlies the *K* allele. The duplication results in two partial *PRLR* genes (that lack both tails), while the *SPEF2* gene remains complete [2]. Incomplete *PRLR* mRNA could be subject to the nonsense-mediated decay mechanism resulting in a lower abundance of *PRLR* mRNA compared to *SPEF2* mRNA. Thus, based on our findings, we believe that, rather than a higher expression of *PRLR*, it is the lack of the C-terminal end of the protein that is responsible for the slow feathering rate at hatch in both chicken and turkey. Interestingly, Nakamura et al. [28] reported that, in a late feathering chicken line, reversion to the fast feathering phenotype occurred in rare instances, but this was not observed in our population.

The *PRLR* mutations in chicken and turkey are clearly independent, but lead to similar phenotypes, which strongly suggests that they have been favoured by identical breeding goals being applied in these two species. Thus, the SF/FF phenotype shows a pattern that is similar to that observed for a small number of monogenic or oligogenic traits under domestication selection, which show independent mutations in the same genes in specific pathways [29]. Coat colour is one of the most common domestication features, which is regulated by a small number of genes (e.g. *KIT*, *MC1R*, and *TYR*) in many domestic animals [30]. Another example in poultry is comb morphology, which is a monogenic trait regulated by the same set of genes but with independent mutations in different breeds (e.g. *EOMES*, *MNR2*, and *SOX5*) [31]. Thus, we hypothesize that the same independent selection applied for domestic feathering rate within and across species has resulted in independent mutations in *PRLR*.

## Conclusions

We describe a case–control GWAS that detected a genomic region on the Z chromosome, which is statistically associated with rate of feathering at hatch in turkey. Within this genomic region, we identified a hemizygous 5-bp frameshift deletion in *PRLR*, which causes the loss of 98 C-terminal AA and is the causal polymorphism for low feathering phenotype in turkey. This is a clear example of similar selection pressures for the same trait (sexing at hatch) in two domestic poultry species that result in two distinct mutations but each affecting the C-terminal end of the same protein, i.e. PRLR. The function of the C-terminal end of this protein, located in the cytoplasm, remains mostly unknown, and further functional studies are necessary to gain more insight in the downstream molecular pathways affected by this mutation.

## Additional files

**Additional file 1:** **Table S1.** Line, animal ID, phenotype, sex and mean sequence coverage for slow feathering (SF) and fast feathering (FF) turkey samples. **Table S2.** Number of variants per type. **Table S3.** Effects of variants per type and region. **Table S4.** Effects of coding SNPs. **Table S5.** List of top 100 significant polymorphisms on chromosome Z.

**Additional file 2:** **Figure S1.** Population PCA for fast feathering (red) and slow-feathering samples (green). **Figure S2:** GWAS results for feathering rate at hatch on chromosome Z. **Figure S3.** Chicken (Galgal) and turkey (Melgal) PRLR protein alignment. PRLR sequences share 90.24% identity. The red (turkey) and blue (chicken) arrows indicate the starting point of the C-terminal loss in PRLR for both species.

**Additional file 3:** CNV-seq results within the associated region on ChrZ:7.9-10.0 Mb for all SF and FF Turkey samples. Each figure shows the log2 ratio between case and control samples in each window across the region.
