# Supplementary material for: Early and late feathering in turkey and chicken: same gene but different mutations
Source: Genet Sel Evol. 2018 Mar 22;50:7. doi: 10.1186/s12711-018-0380-3 (PMC5863816; doi:10.1186/s12711-018-0380-3)
Supplement: Supplementary file 1 — Additional file 1: Table S1. Line, animal ID, phenotype, sex and mean sequence coverage for slow feathering (SF) and fast feathering (FF) turkey samples. Table S2. Number of variants per type. Table S3. Effects of variants per type and region. Table S4. Effects of coding SNPs. Table S5. List of top 100 significant polymorphisms on chromosome Z. [file 12711_2018_380_MOESM1_ESM.pdf]

Table S1: Slow feathering (SF) and fast feathering (FF) turkey samples.

Line	Animal ID	Phenotype	Sex	Mean sequence coverage
H	MG-WUR-121	FF	Female	11.23X
H	MG-WUR-122	FF	Female	9.13X
H	MG-WUR-123	FF	Female	12.43X
H	MG-WUR-124	SF	Female	4.32X
H	MG-WUR-125	SF	Female	16.15X
H	MG-WUR-126	SF	Female	11.54X
H	MG-WUR-127	SF	Female	10.57X
H	MG-WUR-128	FF	Female	17.62X
H	MG-WUR-129	SF	Female	11.79X
H	MG-WUR-130	SF	Female	8.3X
H	MG-WUR-131	SF	Female	6.54X
H	MG-WUR-132	SF	Female	8.93X
H	MG-WUR-133	SF	Female	20.96X
H	MG-WUR-134	SF	Female	9.41X
H	MG-WUR-135	SF	Female	10.68X
H	MG-WUR-136	SF	Female	15.38X
H	MG-WUR-137	FF	Female	2.1X
H	MG-WUR-138	FF	Female	14.14X
H	MG-WUR-139	FF	Female	8.37X
H	MG-WUR-140	FF	Female	11.04X
H	MG-WUR-141	FF	Female	13.21X
H	MG-WUR-142	FF	Female	13.98X
H	MG-WUR-143	FF	Female	8.31X
H	MG-WUR-144	FF	Female	10.89X

Table S2: Variants per type.

Class	Total
SNP	6,595,059
MNP	181,337
INS	737,001
DEL	460,169
MIXED	162,647
**Total**	**8,136,213**

Table S3: Variant effects per type and region.

Type	Count	Percent
**3 prime UTR variant**	294,003	0.893%
**5 prime UTR premature start codon gain variant**	13,260	0.04%
**5 prime UTR variant**	84,860	0.258%
**bidirectional gene fusion**	16	0%
**conservative inframe deletion**	523	0.002%
**conservative inframe insertion**	337	0.001%
**disruptive inframe deletion**	299	0.001%
**disruptive inframe insertion**	189	0.001%
**downstream gene variant**	3,536,804	10.739%
**frameshift variant**	3,435	0.01%
**gene fusion**	40	0%
**initiator codon variant**	21	0%
**intergenic region**	3,914,527	11.886%
**intragenic variant**	819,407	2.488%
**intron variant**	10,462,820	31.77%
**missense variant**	80,888	0.246%
**non coding transcript exon variant**	365,873	1.111%
**non coding transcript variant**	9,645,839	29.289%
**splice acceptor variant**	1,563	0.005%
**splice donor variant**	1,721	0.005%
**splice region variant**	47,083	0.143%
**start lost**	368	0.001%
**stop gained**	790	0.002%
**stop lost**	160	0%
**stop retained variant**	97	0%
**synonymous variant**	140,703	0.427%
**upstream gene variant**	3,517,777	10.681%

Table S4: Effect of coding SNPs. The missense / silent ratio: 0.545.

Type	Count	Percent
**MISSENSE**	76,603	35.184%
**NONSENSE**	691	0.317%
**SILENT**	140,426	64.498%

Table S5: List of top 100 significant polymorphisms.

Chromosome	Pos	Ref. allele	SF carrier freq.	FF carrier freq	Alt. allele	P-val	Protein altering
ChrZ	8857275	T	1	0.09091	C	4.12E-11	NO
ChrZ	9185457	T	0	0.9091	G	4.12E-11	NO
ChrZ	8642592	T	1	0.1364	C	3.71E-10	NO
ChrZ	9019282	A	0	0.8636	G	3.71E-10	NO
ChrZ	9040199	A	0	0.8636	G	3.71E-10	NO
ChrZ	9040239	T	0	0.8636	A	3.71E-10	NO
ChrZ	8707888	G	0	0.8333	T	1.27E-09	NO
ChrZ	8964132	G	1	0.1667	A	1.27E-09	NO
ChrZ	8964152	A	1	0.1667	G	1.27E-09	NO
ChrZ	9011850	G	0	0.8333	A	1.27E-09	NO
ChrZ	8092917	C	1	0.1364	T	2.19E-09	NO
ChrZ	9007699	T	1	0.1364	C	2.19E-09	NO
**ChrZ**	**9426018**	**GT**	**1**	**0.1818**	**GTTGGTT**	**2.60E-09**	**YES (PRLR)**
ChrZ	8342756	A	1	0.1818	T	2.60E-09	NO
ChrZ	8351160	C	0	0.8182	T	2.60E-09	NO
ChrZ	8390359	AG	0	0.8182	AGG	2.60E-09	NO
ChrZ	8448741	T	0	0.8182	C	2.60E-09	NO
ChrZ	8735559	A	1	0.1818	C	2.60E-09	NO
ChrZ	8824038	G	0	0.8182	A	2.60E-09	NO
ChrZ	8824824	C	1	0.1818	T	2.60E-09	NO
ChrZ	8831283	C	0	0.8182	T	2.60E-09	NO
ChrZ	8838674	A	0	0.8182	G	2.60E-09	NO
ChrZ	8851245	A	1	0.1818	C	2.60E-09	NO
ChrZ	8852149	A	1	0.1818	G	2.60E-09	NO
ChrZ	8856691	A	1	0.1818	G	2.60E-09	NO
ChrZ	8857364	C	0	0.8182	A	2.60E-09	NO
ChrZ	8953215	A	1	0.1818	G	2.60E-09	NO
ChrZ	8957954	T	0	0.8182	A	2.60E-09	NO
ChrZ	8962958	C	1	0.1818	T	2.60E-09	NO
ChrZ	8965325	T	1	0.1818	A	2.60E-09	NO
ChrZ	8973222	C	1	0.1818	A	2.60E-09	NO
ChrZ	8978769	T	0	0.8182	C	2.60E-09	NO
ChrZ	8981361	A	0	0.8182	G	2.60E-09	NO
ChrZ	8983221	A	1	0.1818	G	2.60E-09	NO
ChrZ	8985961	G	1	0.1818	A	2.60E-09	NO
ChrZ	8991818	G	1	0.1818	T	2.60E-09	NO
ChrZ	8993640	G	0	0.8182	A	2.60E-09	NO
ChrZ	8997377	G	0	0.8182	A	2.60E-09	NO
ChrZ	8997673	C	0	0.8182	G	2.60E-09	NO
ChrZ	9000514	G	1	0.1818	A	2.60E-09	NO
ChrZ	9000640	CTTTTTTTTTC	0	0.8182	CTTTTTTTTC	2.60E-09	NO
ChrZ	9001463	G	0	0.8182	A	2.60E-09	NO
ChrZ	9001604	T	1	0.1818	C	2.60E-09	NO
ChrZ	9001884	T	0	0.8182	C	2.60E-09	NO
ChrZ	9002293	A	0	0.8182	G	2.60E-09	NO
ChrZ	9002479	T	0	0.8182	C	2.60E-09	NO
ChrZ	9002550	T	1	0.1818	C	2.60E-09	NO
ChrZ	9003502	A	0	0.8182	T	2.60E-09	YES (ADAMTS12)
ChrZ	9005712	G	1	0.1818	A	2.60E-09	NO
ChrZ	9006017	T	1	0.1818	C	2.60E-09	NO
ChrZ	9008586	A	1	0.1818	G	2.60E-09	NO
ChrZ	9008937	C	1	0.1818	T	2.60E-09	NO
ChrZ	9009057	C	1	0.1818	T	2.60E-09	NO
ChrZ	9011233	TT	0	0.8182	TGT	2.60E-09	NO
ChrZ	9012253	G	0	0.8182	A	2.60E-09	NO
ChrZ	9012757	A	0	0.8182	G	2.60E-09	NO
ChrZ	9014146	A	0	0.8182	C	2.60E-09	NO
ChrZ	9014751	G	0	0.8182	A	2.60E-09	NO
ChrZ	9017562	C	0	0.8182	T	2.60E-09	NO
ChrZ	9017679	A	0	0.8182	G	2.60E-09	NO
ChrZ	9017967	T	0	0.8182	C	2.60E-09	NO
ChrZ	9018427	A	0	0.8182	G	2.60E-09	NO
ChrZ	9021484	A	0	0.8182	G	2.60E-09	NO
ChrZ	9022805	G	0	0.8182	A	2.60E-09	NO

ChrZ	9024002	A	0	0.8182	G	2.60E-09	NO
ChrZ	9024415	ATTTTTTTTTG	0	0.8182	ATTTTTTTTG	2.60E-09	NO
ChrZ	9024917	G	1	0.1818	A	2.60E-09	NO
ChrZ	9032079	G	0	0.8182	A	2.60E-09	NO
ChrZ	9032522	AGT	0	0.8182	AGGT	2.60E-09	NO
ChrZ	9044048	C	0	0.8182	T	2.60E-09	NO
ChrZ	9049191	T	0	0.8182	C	2.60E-09	NO
ChrZ	9050159	T	0	0.8182	A	2.60E-09	NO
ChrZ	9050501	G	0	0.8182	A	2.60E-09	NO
ChrZ	9050750	A	0	0.8182	G	2.60E-09	NO
ChrZ	9055326	T	1	0.1818	G	2.60E-09	NO
ChrZ	9055593	G	0	0.8182	C	2.60E-09	NO
ChrZ	9055595	A	0	0.8182	C	2.60E-09	NO
ChrZ	9055839	A	0	0.8182	G	2.60E-09	NO
ChrZ	9057437	G	0	0.8182	A	2.60E-09	NO
ChrZ	9057946	G	1	0.1818	C	2.60E-09	NO
ChrZ	9091728	G	0	0.8182	T	2.60E-09	NO
ChrZ	9095264	C	0	0.8182	T	2.60E-09	NO
ChrZ	9136697	C	0	0.8182	T	2.60E-09	NO
ChrZ	9160481	A	0	0.8182	G	2.60E-09	NO
ChrZ	9181053	T	0	0.8182	C	2.60E-09	NO
ChrZ	9181900	T	1	0.1818	A	2.60E-09	NO
ChrZ	9200910	T	0	0.8182	C	2.60E-09	NO
ChrZ	9790932	T	1	0.1818	C	2.60E-09	NO
ChrZ	9052821	C	0	0.9	A	3.35E-09	NO
ChrZ	8986123	TGTGAG	0.04167	0.8636	TGTGAGTGAG	8.53E-09	NO
ChrZ	8755552	T	1	0.2	C	1.16E-08	NO
ChrZ	8857343	T	0	0.8	C	1.16E-08	NO
ChrZ	8985758	T	0	0.8	C	1.16E-08	NO
ChrZ	9014562	A	0	0.8	G	1.16E-08	NO
ChrZ	9024781	T	0	0.8	C	1.16E-08	NO
ChrZ	9041949	A	0	0.8	G	1.16E-08	NO
ChrZ	9421693	G	1	0.2	A	1.16E-08	NO
ChrZ	8857649	C	0.08333	0.9091	G	1.38E-08	NO
ChrZ	7957723	T	1	0.1818	C	1.42E-08	NO
