# Supplementary material for: Early and late feathering in turkey and chicken: same gene but different mutations
Source: Genet Sel Evol. 2018 Mar 22;50:7. doi: 10.1186/s12711-018-0380-3 (PMC5863816; doi:10.1186/s12711-018-0380-3)
Supplement: Supplementary file 2 — Additional file 2: Figure S1. Population PCA for fast feathering (red) and slow-feathering samples (green). Figure S2: GWAS results for feathering rate at hatch on chromosome Z. Figure S3. Chicken (Galgal) and turkey (Melgal) PRLR protein alignment. PRLR sequences share 90.24% identity. The red (turkey) and blue (chicken) arrows indicate the starting point of the C-terminal loss in PRLR for both species. [file 12711_2018_380_MOESM2_ESM.pdf]

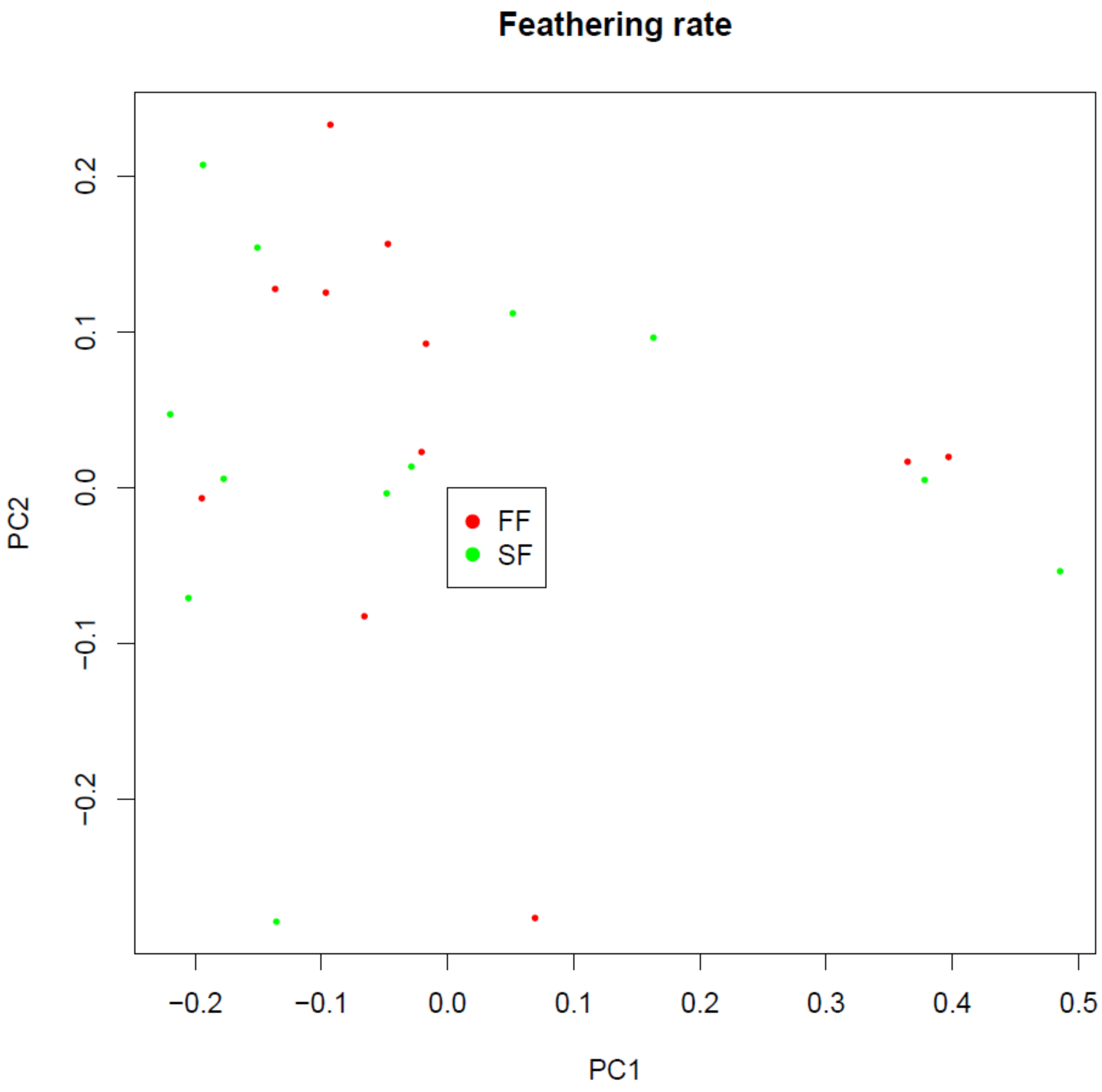

Figure S1: Population PCA for fast feathering (red) and slow-feathering samples (green).

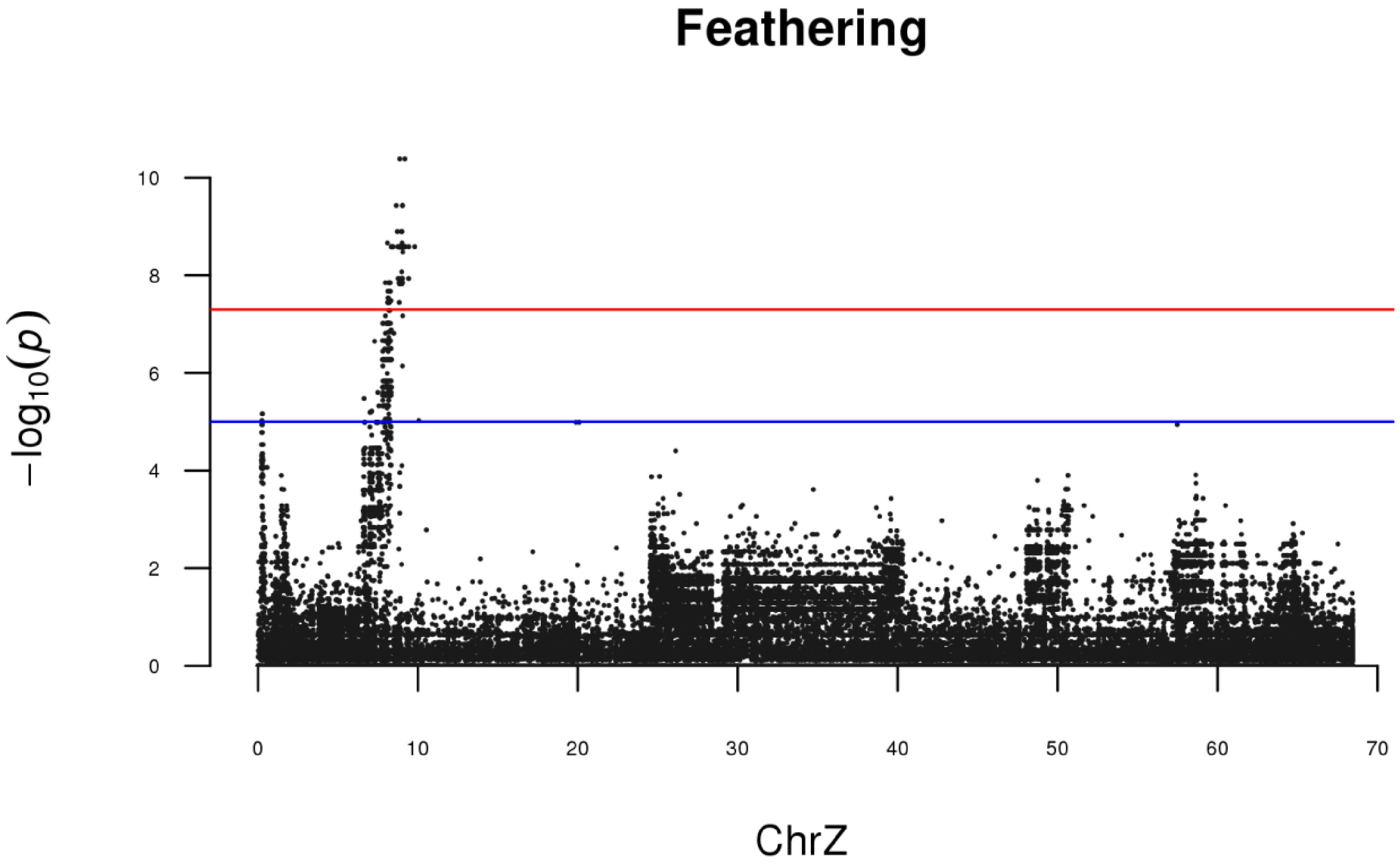

Figure S2: GWAS results for feathering rate at hatch on chromosome Z.

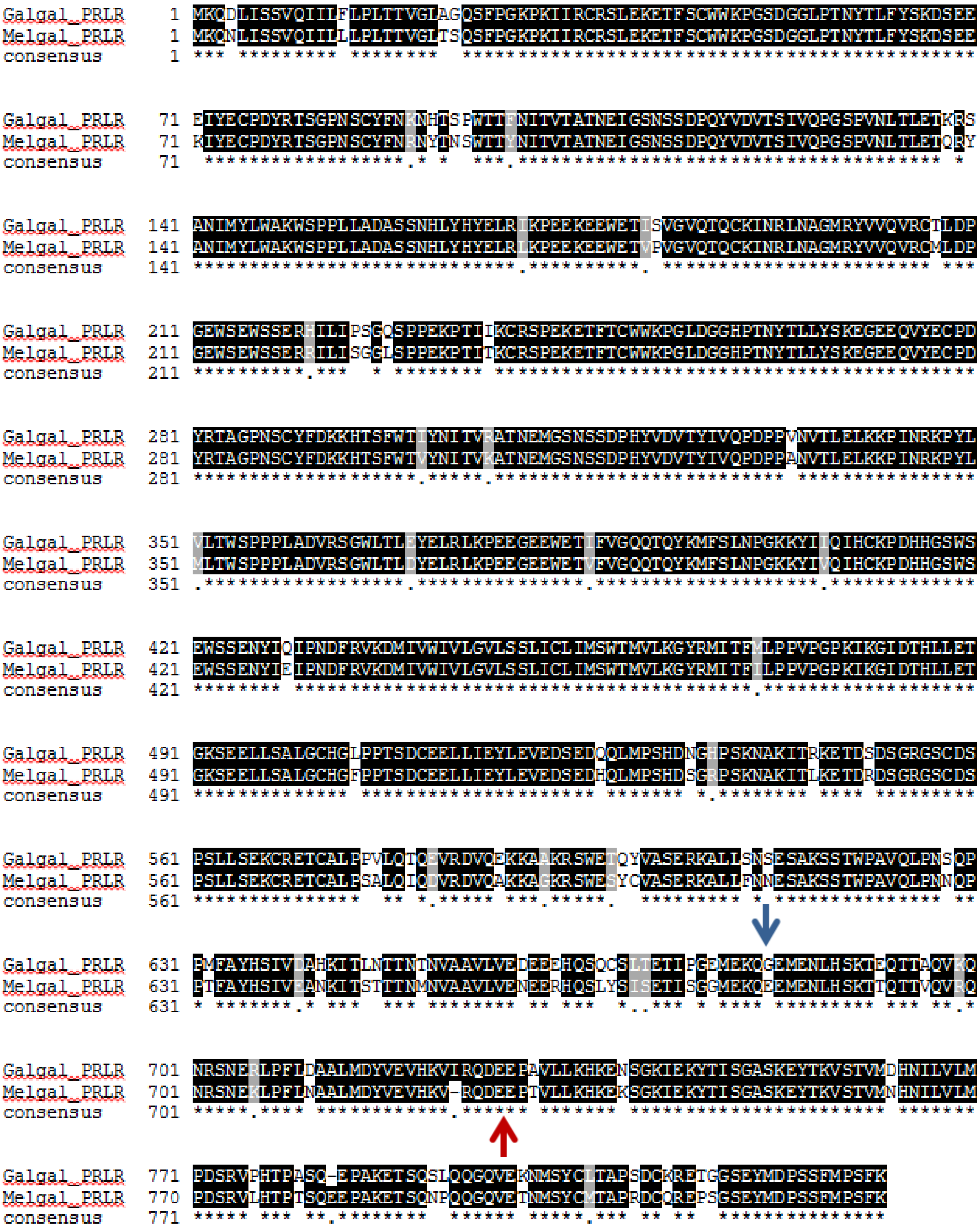

Figure S3: Chicken (Galgal) and turkey (Melgal) *PRLR* protein alignment. Sequences share 90.24 percent identity. The red (turkey) and blue (chicken) arrows indicate the start location of the C-terminal loss in both species.
