# Supplementary material for: Early and late feathering in turkey and chicken: same gene but different mutations
Source: Genet Sel Evol. 2018 Mar 22;50:7. doi: 10.1186/s12711-018-0380-3 (PMC5863816; doi:10.1186/s12711-018-0380-3)
Supplement: Supplementary file 3 — Additional file 3: CNV-seq results within the associated region on ChrZ:7.9-10.0 Mb for all SF and FF Turkey samples. Each figure shows the log2 ratio between case and control samples in each window across the region. [file 12711_2018_380_MOESM3_ESM.pdf]

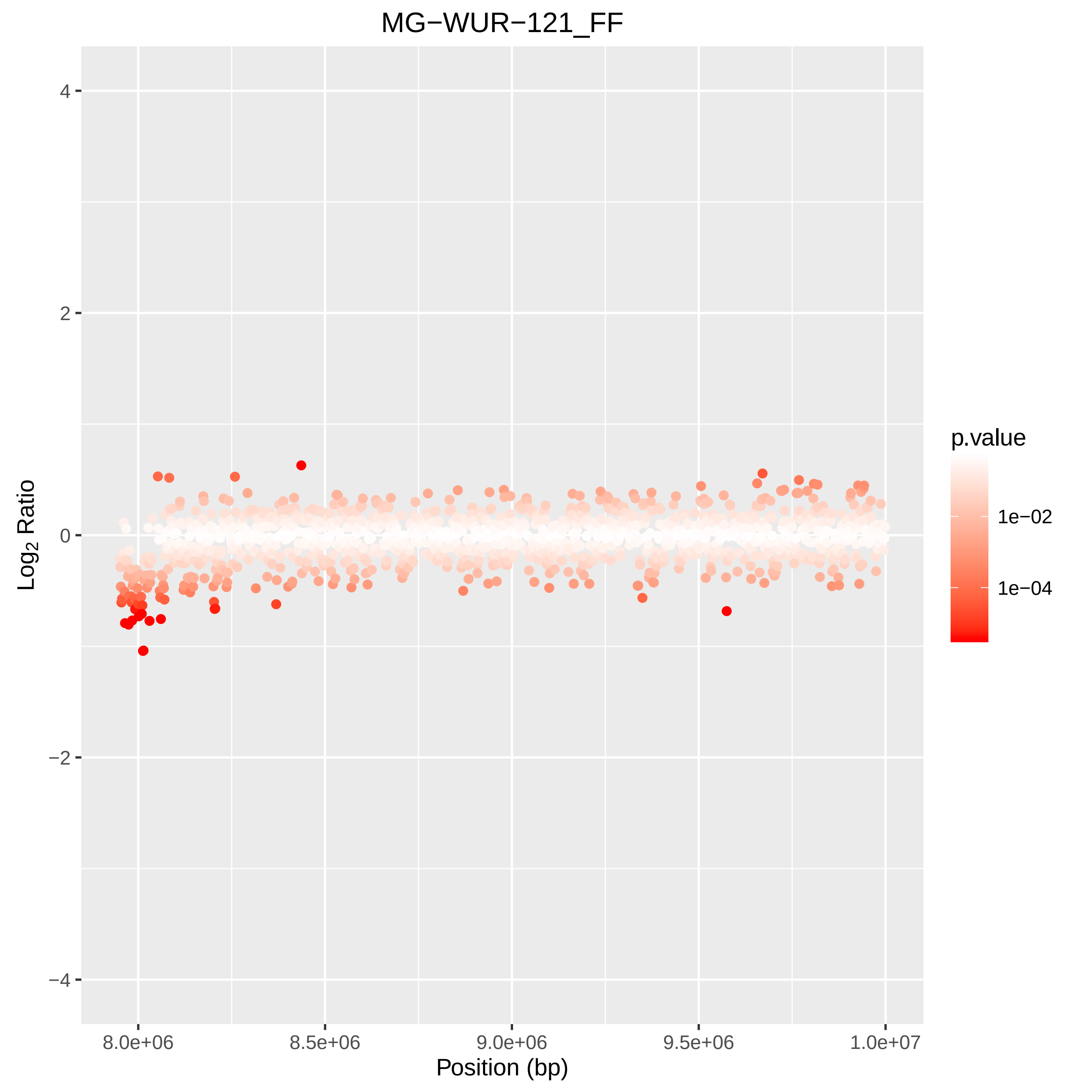

MG−WUR−121_FF
Log2 Ratio
4
2
0
−2
−4
8.0e+06
8.5e+06
9.0e+06
9.5e+06
1.0e+07
Position (bp)
p.value
1e−02
1e−04

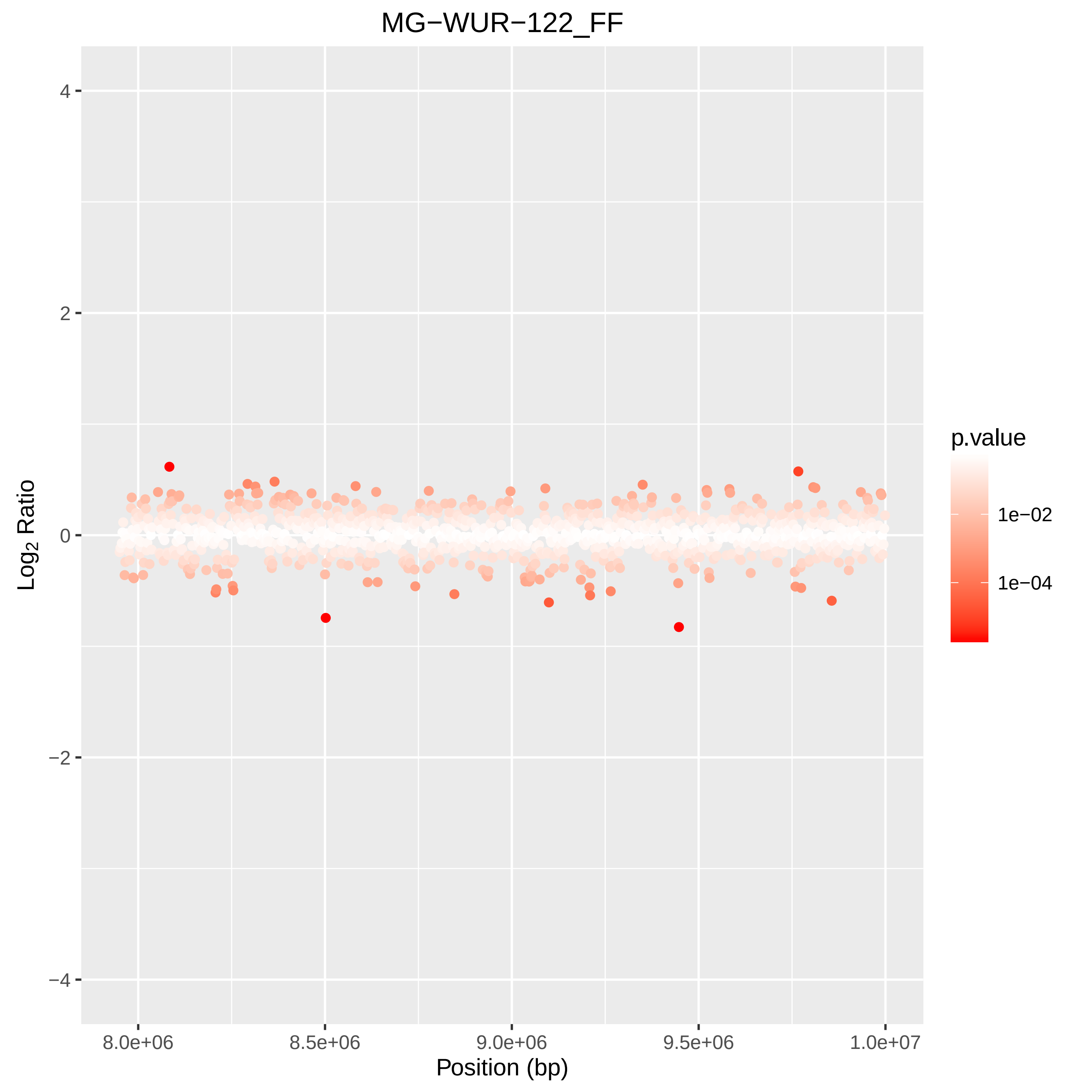

MG−WUR−122_FF
4
2
0
−2
−4
Log2 Ratio
8.0e+06
8.5e+06
9.0e+06
9.5e+06
1.0e+07
Position (bp)
p.value
1e−02
1e−04

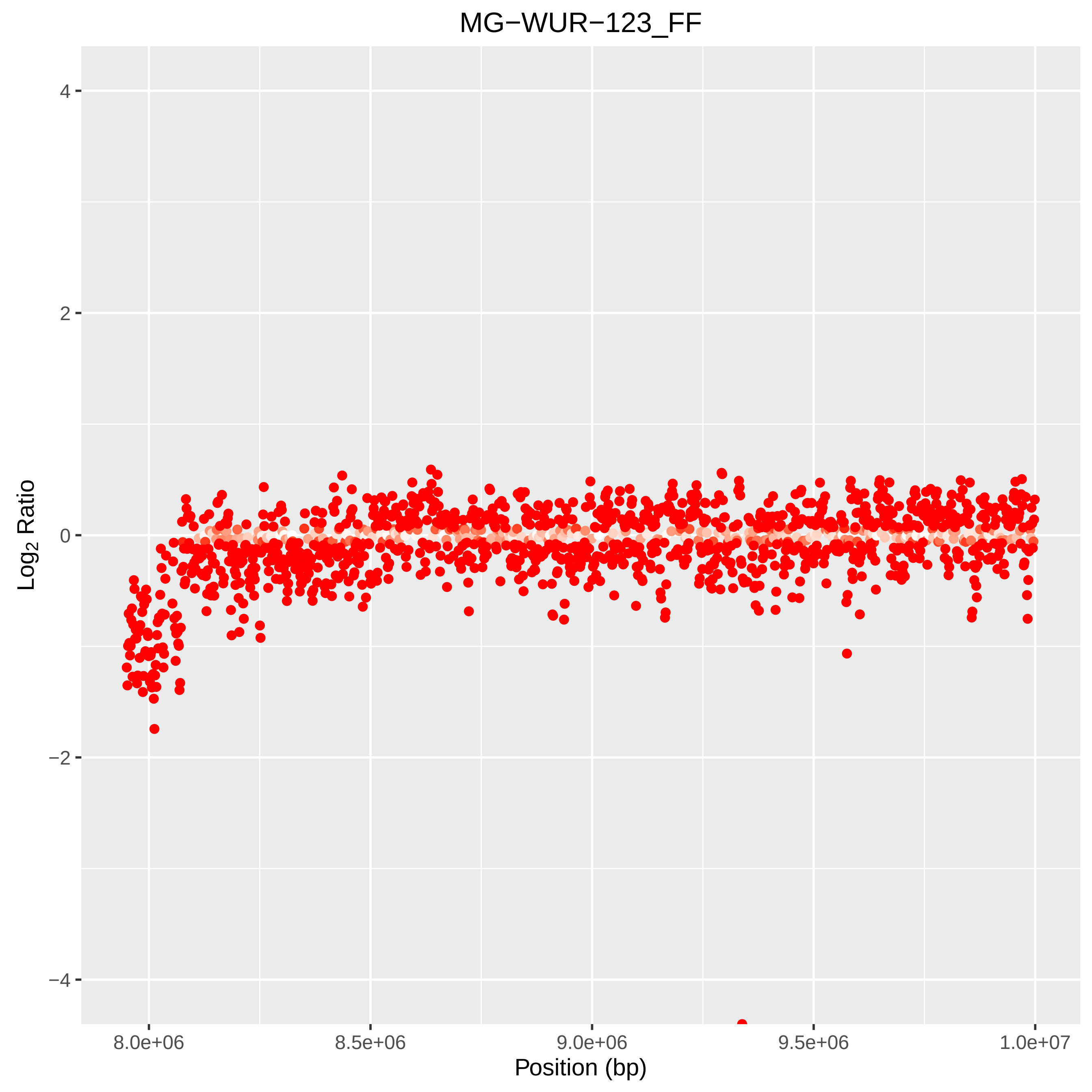

MG−WUR−123_FF
Log2 Ratio
Position (bp)
4
2
0
−2
−4
8.0e+06
8.5e+06
9.0e+06
9.5e+06
1.0e+07

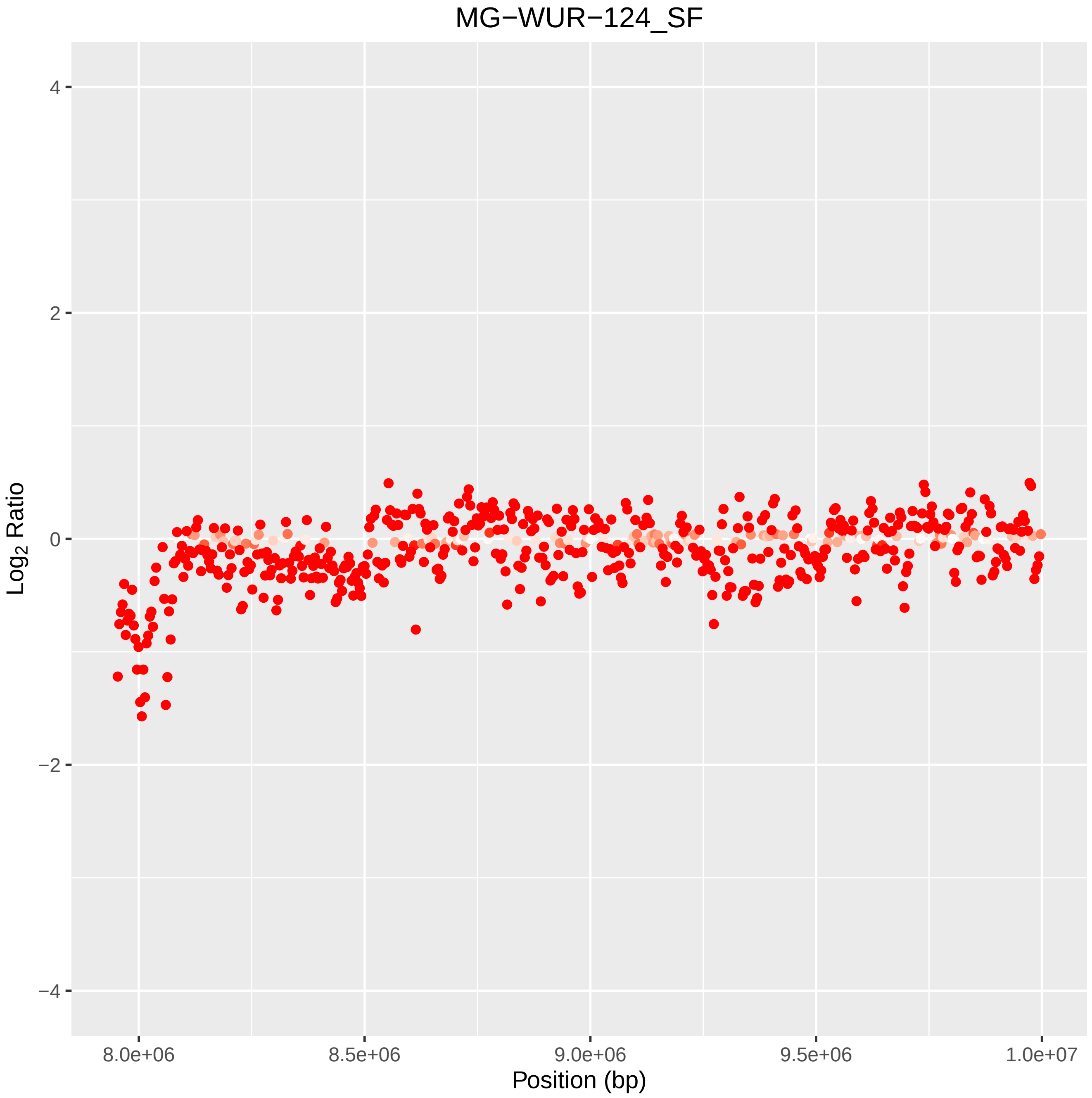

MG−WUR−124_SF
Log2 Ratio
4
2
0
−2
−4
8.0e+06
8.5e+06
9.0e+06
9.5e+06
1.0e+07
Position (bp)

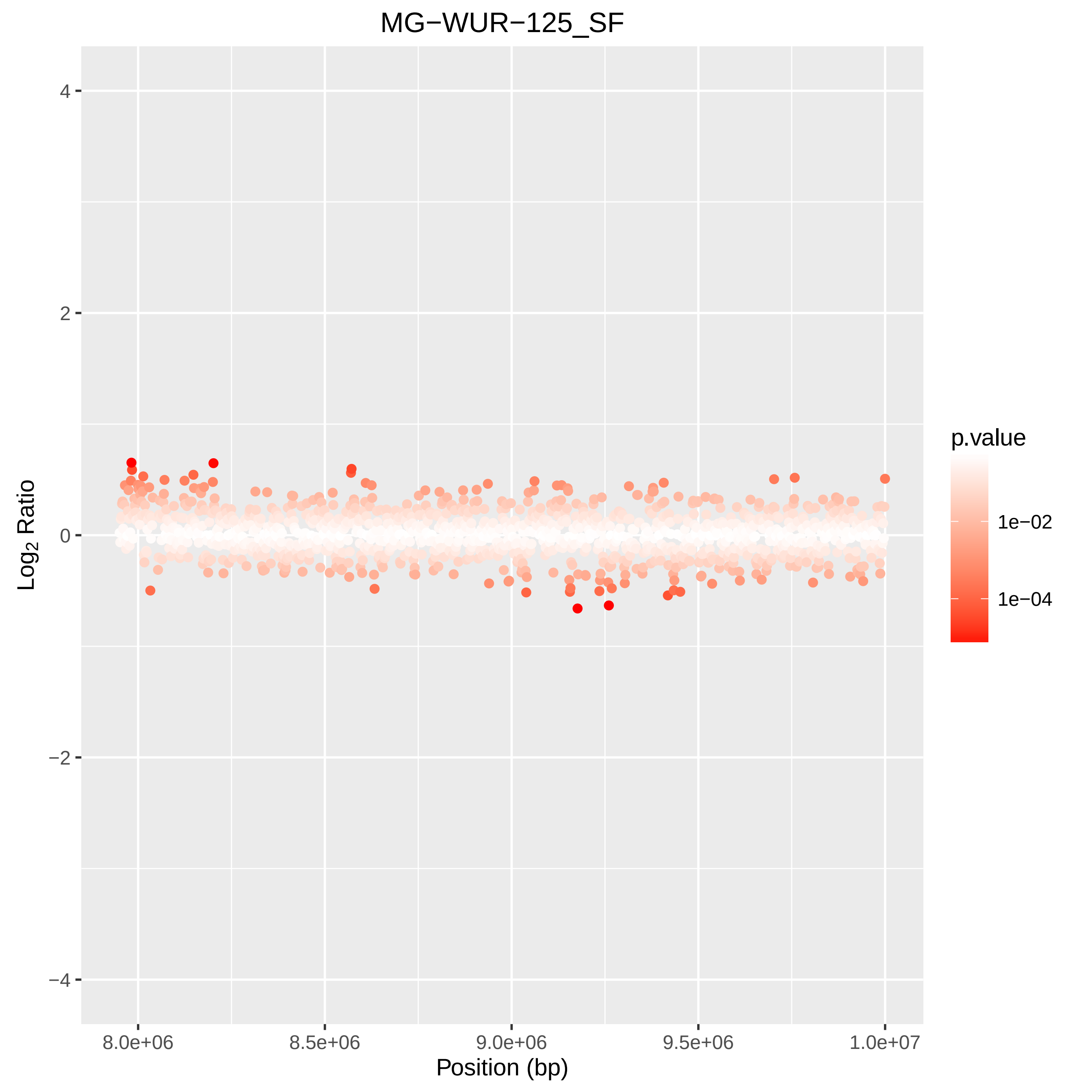

MG−WUR−125_SF
Log2 Ratio
4
2
0
−2
−4
8.0e+06
8.5e+06
9.0e+06
9.5e+06
1.0e+07
Position (bp)
p.value
1e−02
1e−04

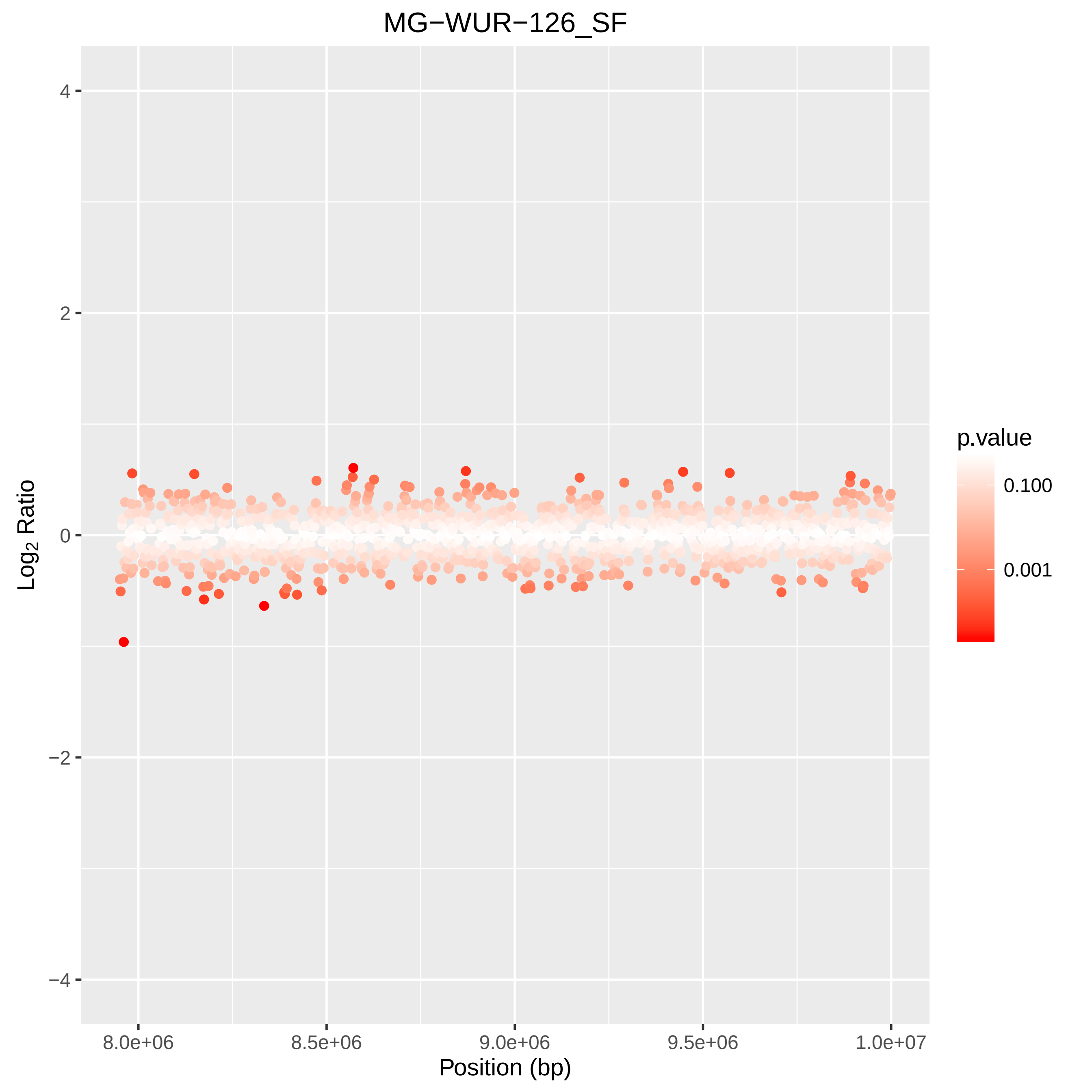
MG−WUR−126_SF
Log2 Ratio
4
2
0
−2
−4
Position (bp)
8.0e+06
8.5e+06
9.0e+06
9.5e+06
1.0e+07
p.value
0.100
0.001

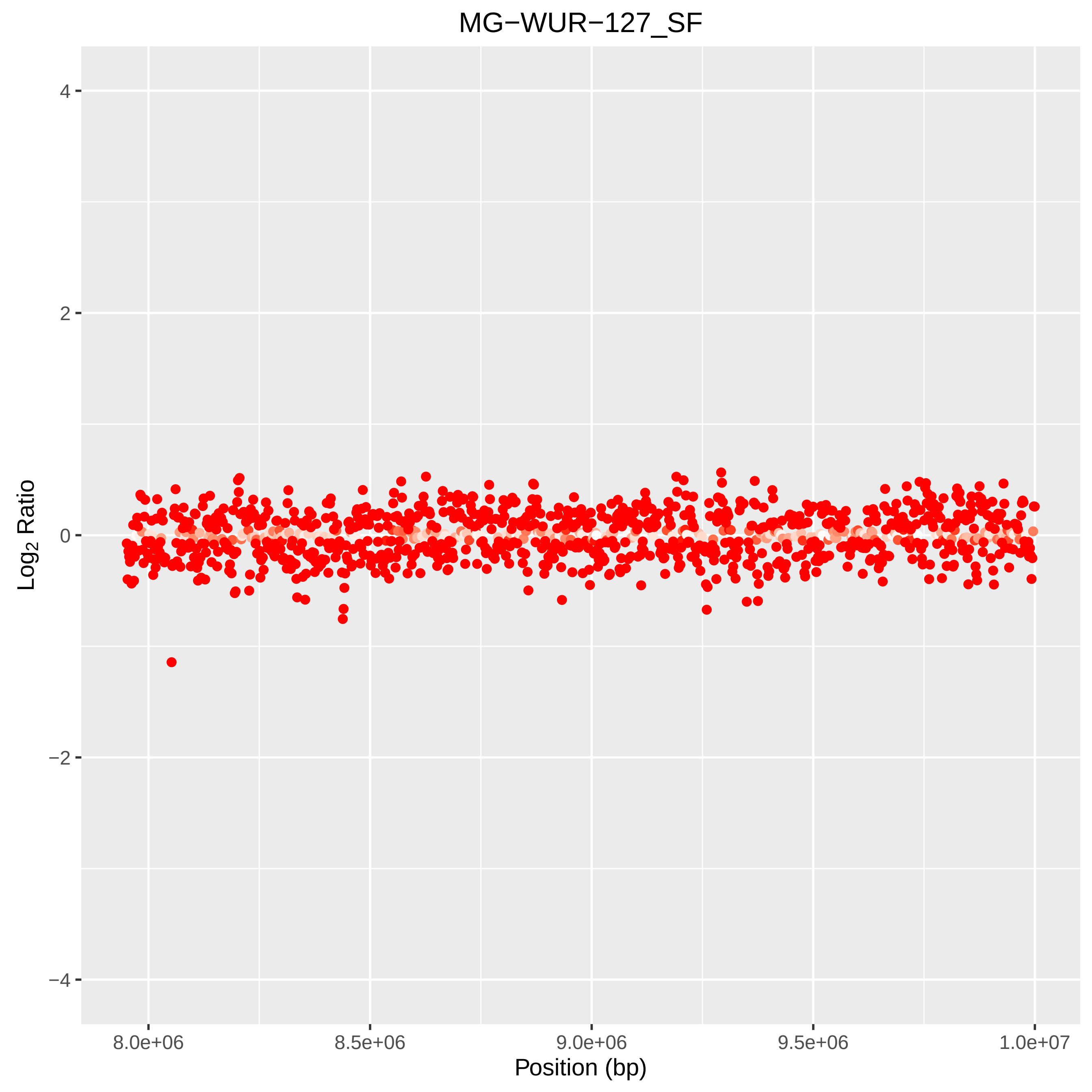

MG−WUR−127_SF
Log2 Ratio
4
2
0
−2
−4
8.0e+06
8.5e+06
9.0e+06
9.5e+06
1.0e+07
Position (bp)

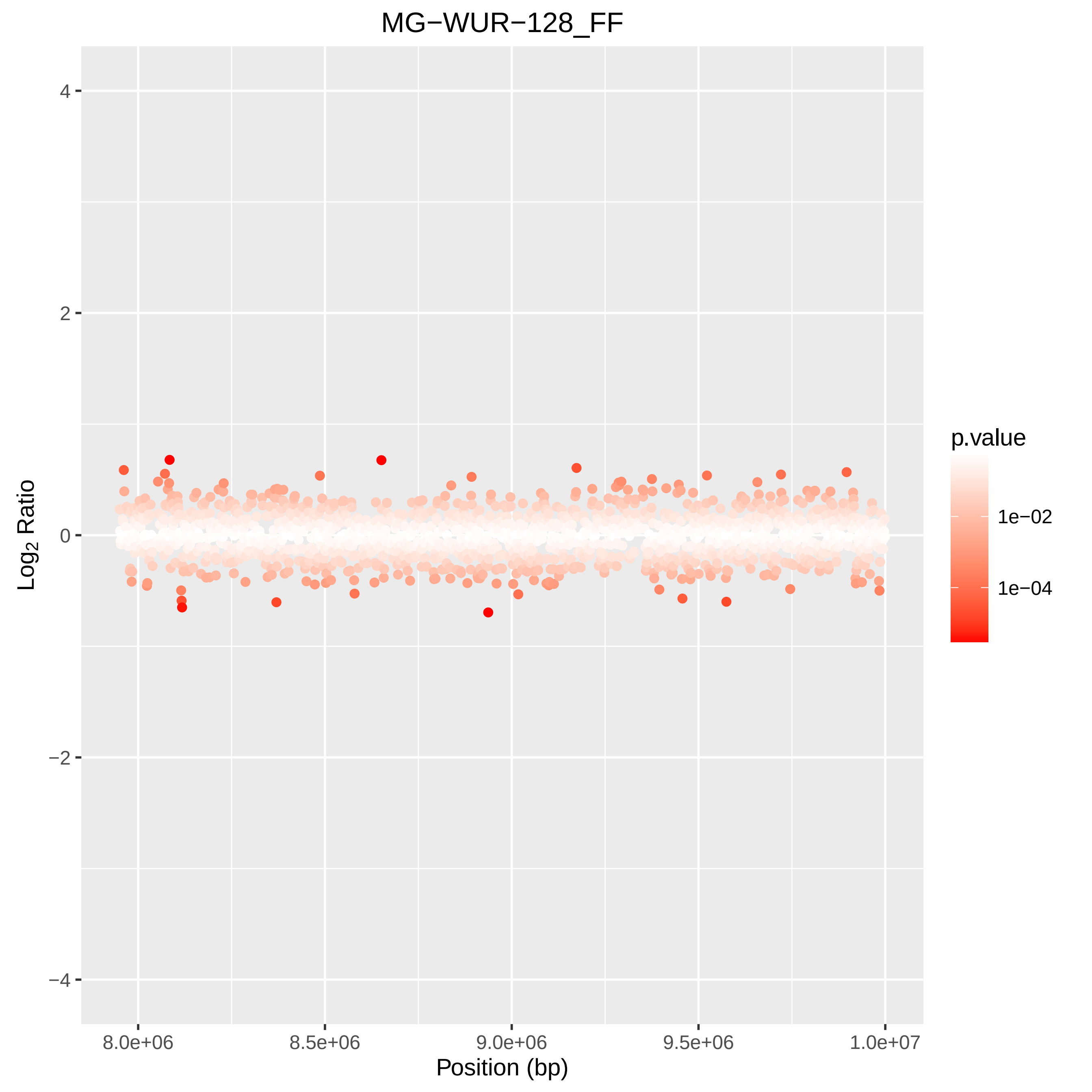

MG−WUR−128_FF
4
2
0
−2
−4
Log2 Ratio
8.0e+06
8.5e+06
9.0e+06
9.5e+06
1.0e+07
Position (bp)
p.value
1e−02
1e−04

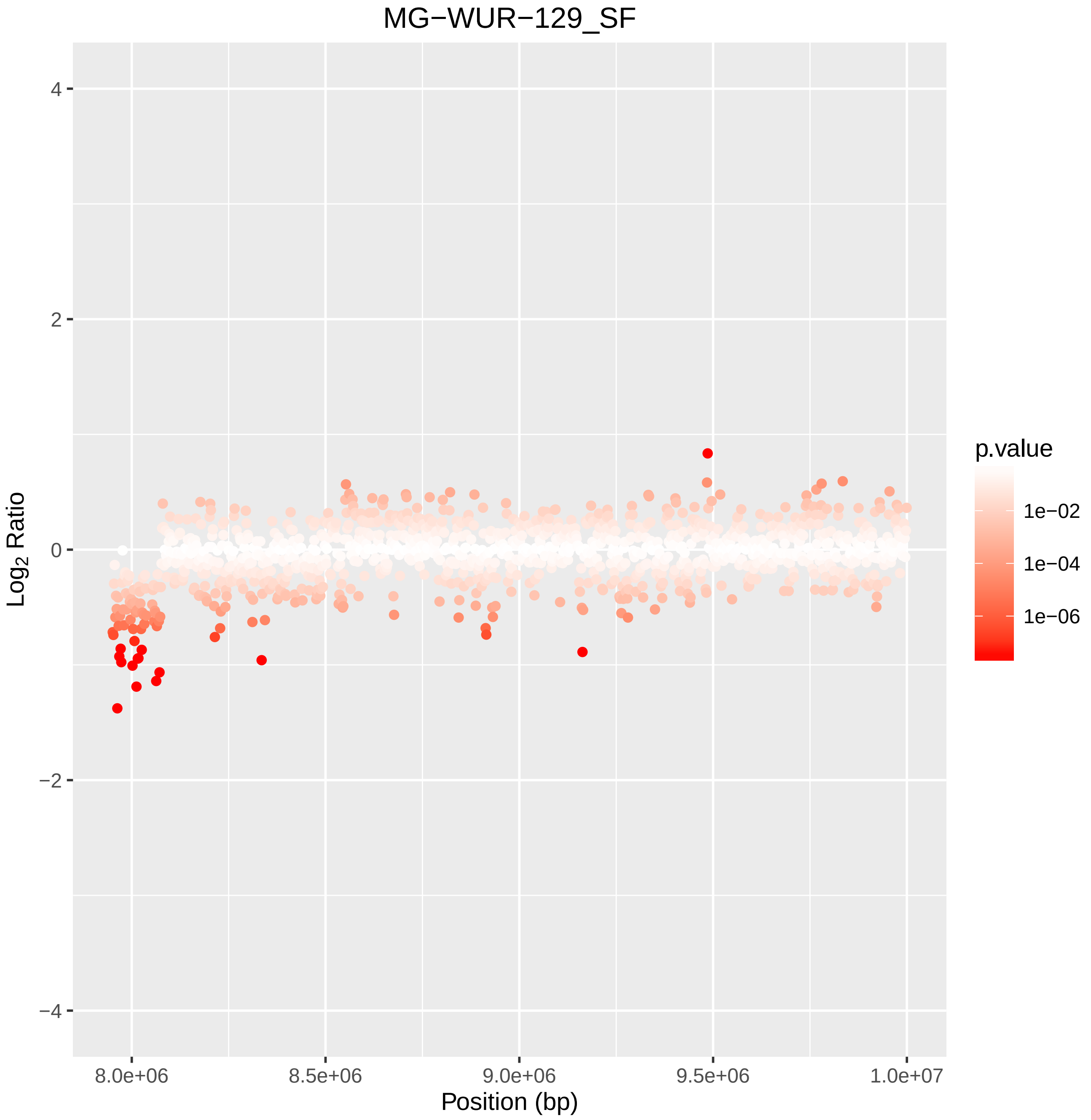
MG−WUR−129_SF
Log2 Ratio
4
2
0
−2
−4
8.0e+06
8.5e+06
9.0e+06
9.5e+06
1.0e+07
Position (bp)
p.value
1e−02
1e−04
1e−06

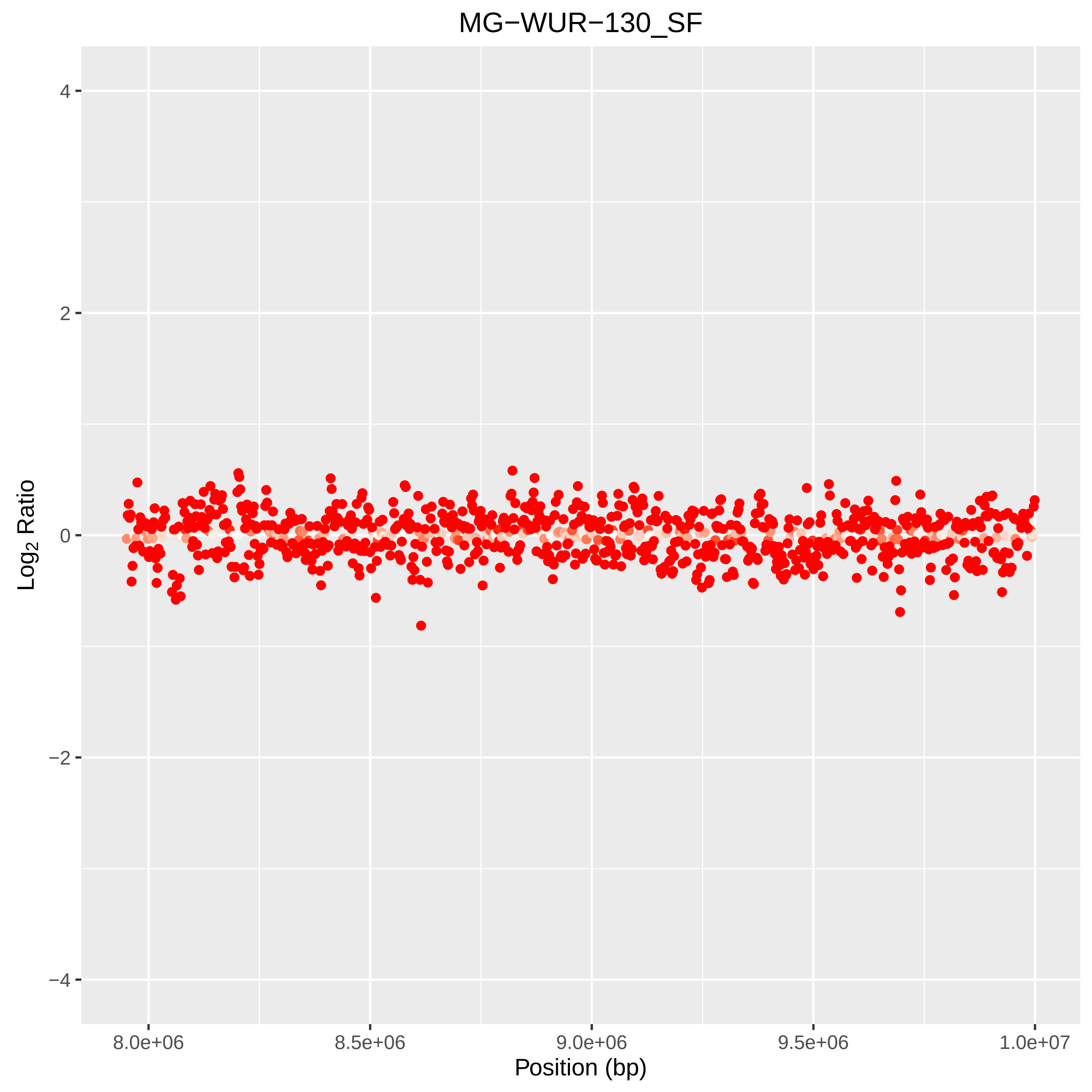

MG−WUR−130_SF
Log2 Ratio
4
2
0
−2
−4
8.0e+06
8.5e+06
9.0e+06
9.5e+06
1.0e+07
Position (bp)

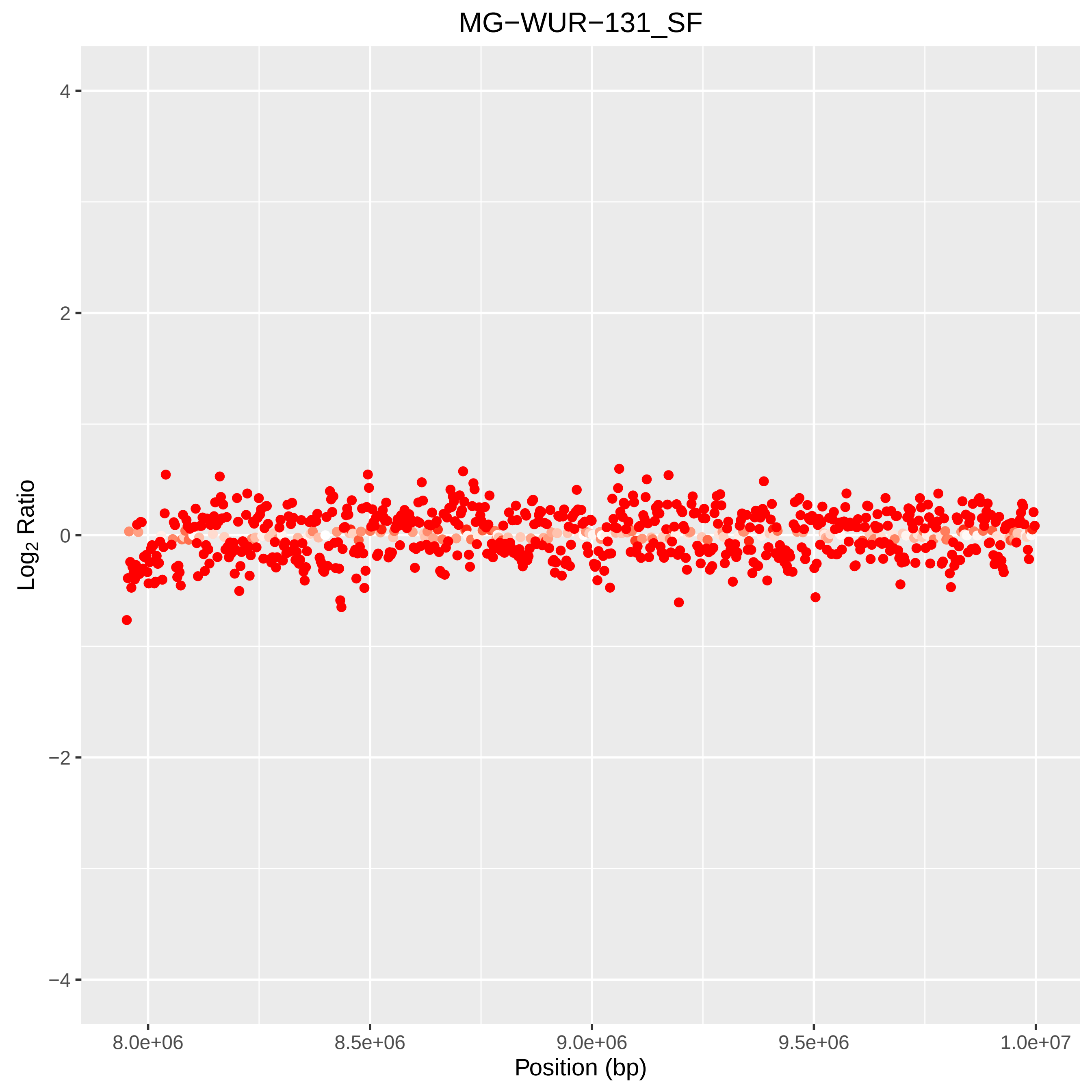
MG−WUR−131_SF
Log2 Ratio
4
2
0
−2
−4
8.0e+06
8.5e+06
9.0e+06
9.5e+06
1.0e+07
Position (bp)

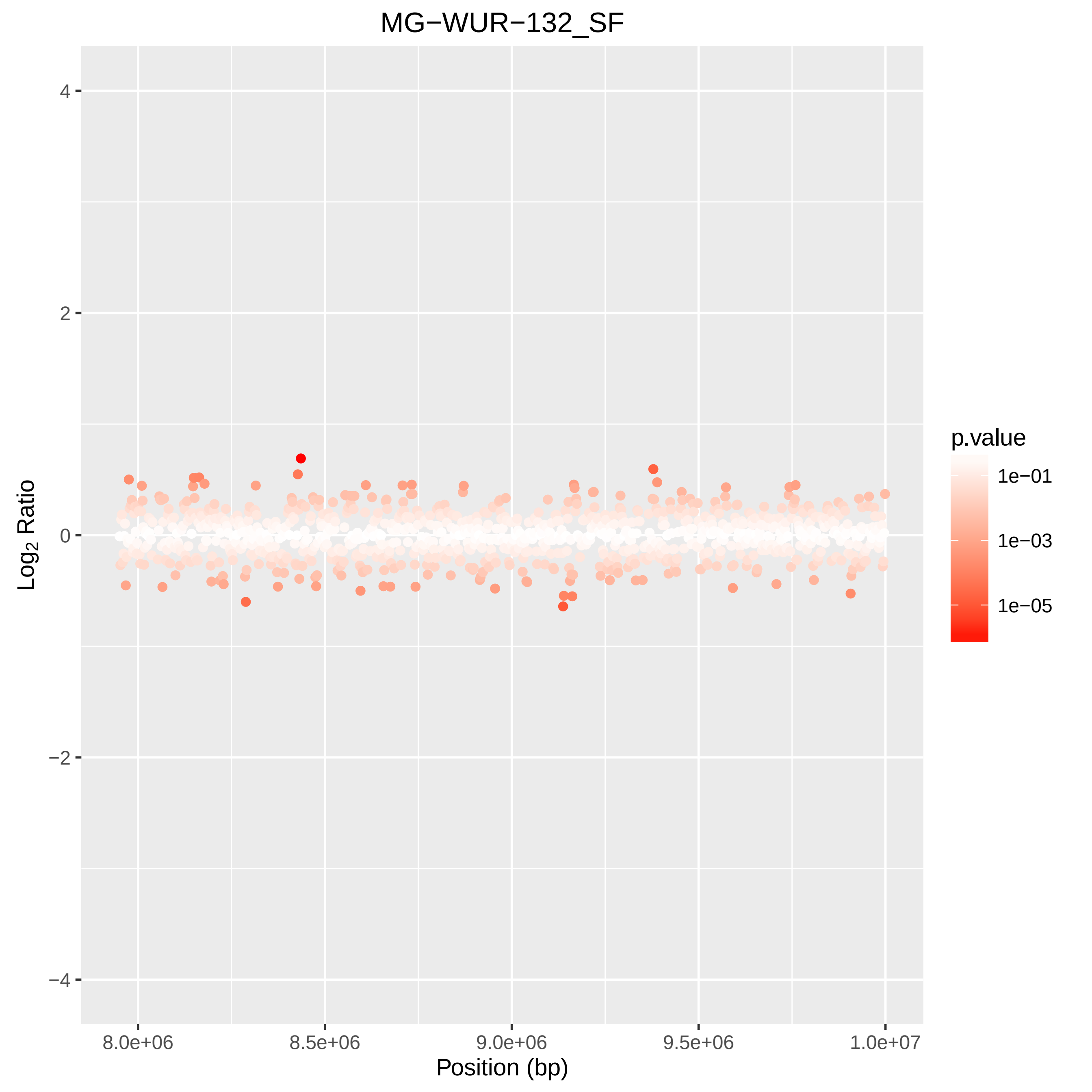
MG−WUR−132_SF
Log2 Ratio
Position (bp)
4
2
0
−2
−4
8.0e+06
8.5e+06
9.0e+06
9.5e+06
1.0e+07
p.value
1e−01
1e−03
1e−05

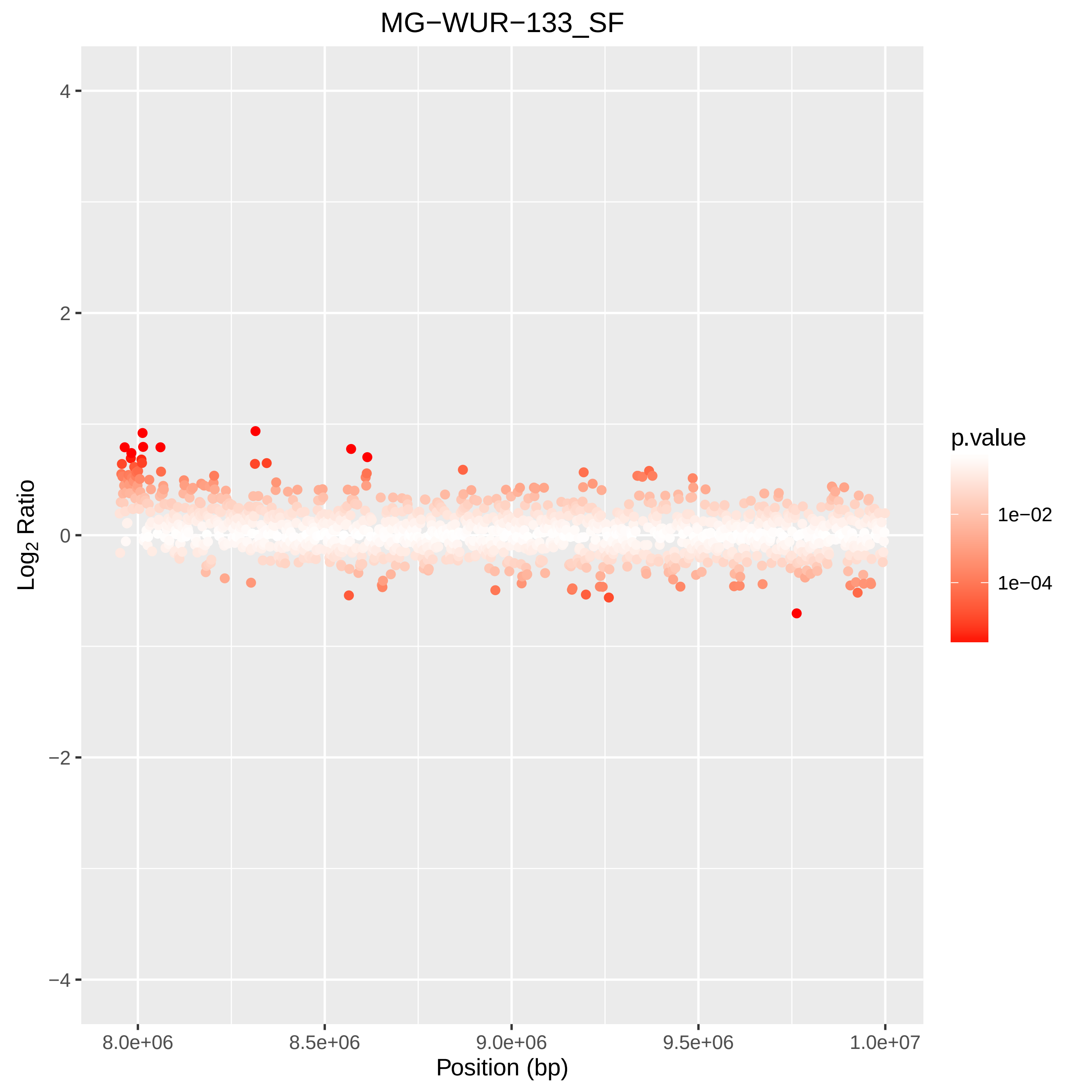

MG−WUR−133_SF
Log2 Ratio
Position (bp)
p.value
1e−02
1e−04
4
2
0
−2
−4
8.0e+06
8.5e+06
9.0e+06
9.5e+06
1.0e+07

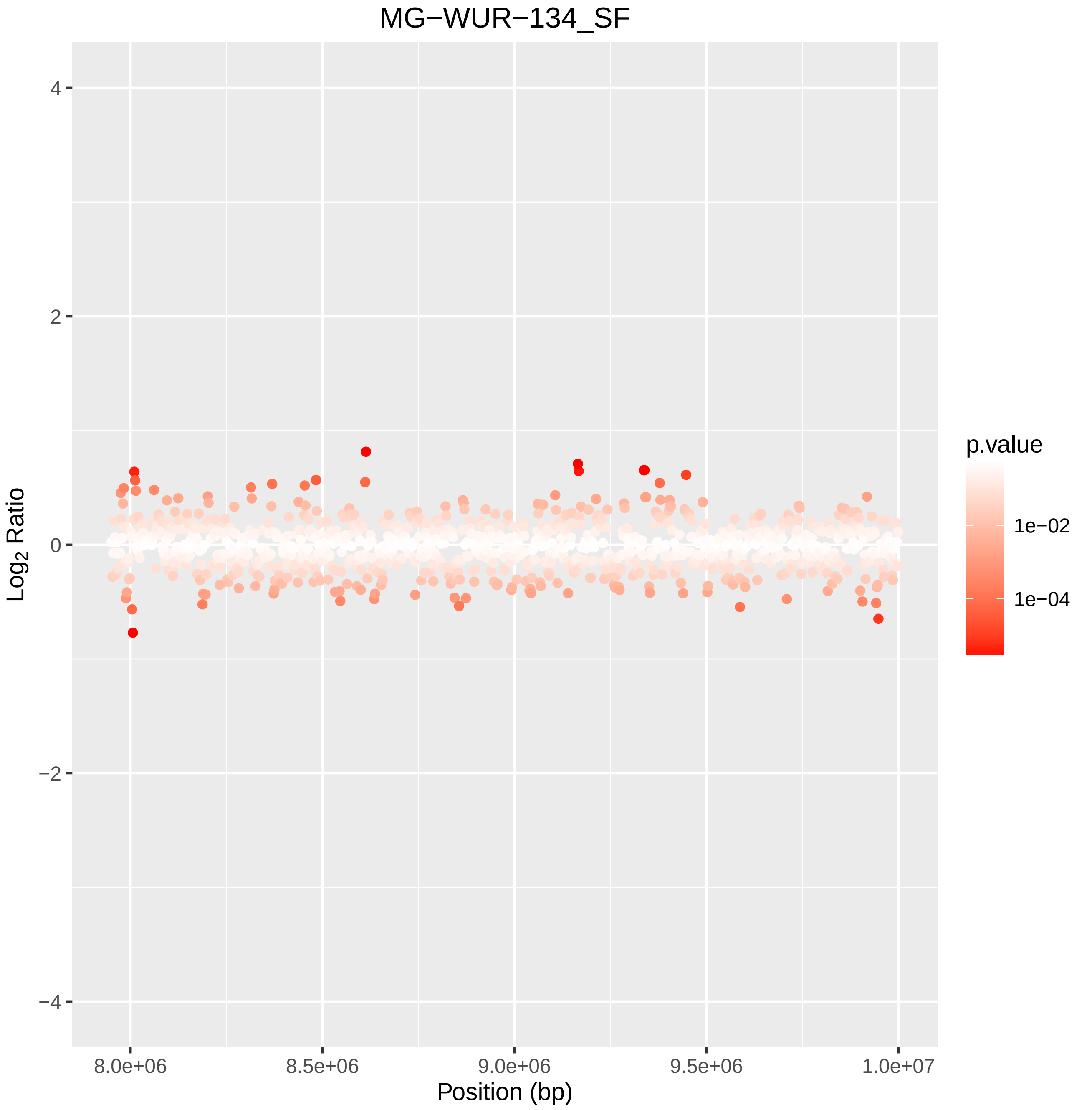

MG−WUR−134_SF
Log2 Ratio
4
2
0
−2
−4
8.0e+06
8.5e+06
9.0e+06
9.5e+06
1.0e+07
Position (bp)
p.value
1e−02
1e−04

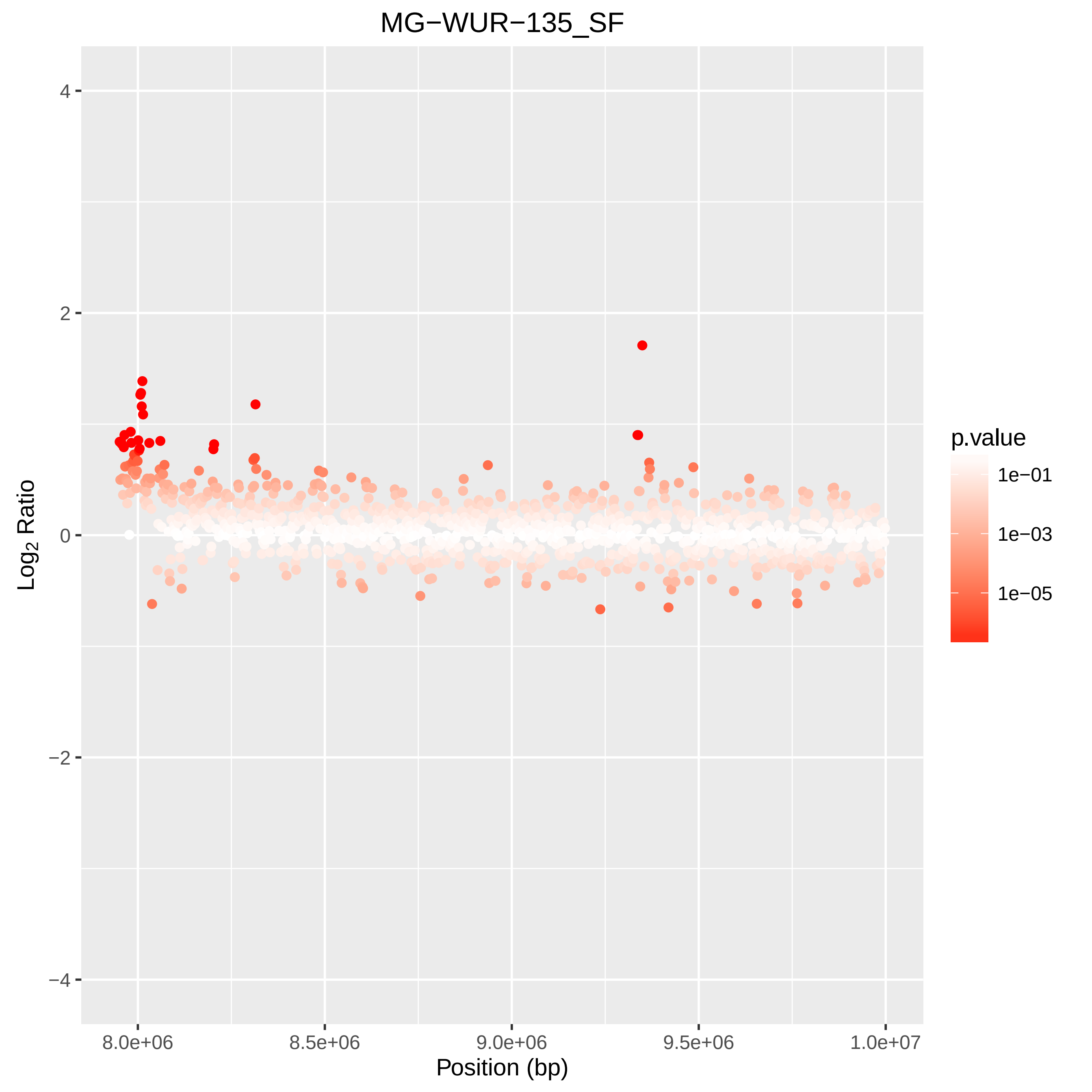

MG−WUR−135_SF
Log2 Ratio
4
2
0
−2
−4
8.0e+06
8.5e+06
9.0e+06
9.5e+06
1.0e+07
Position (bp)
p.value
1e−01
1e−03
1e−05

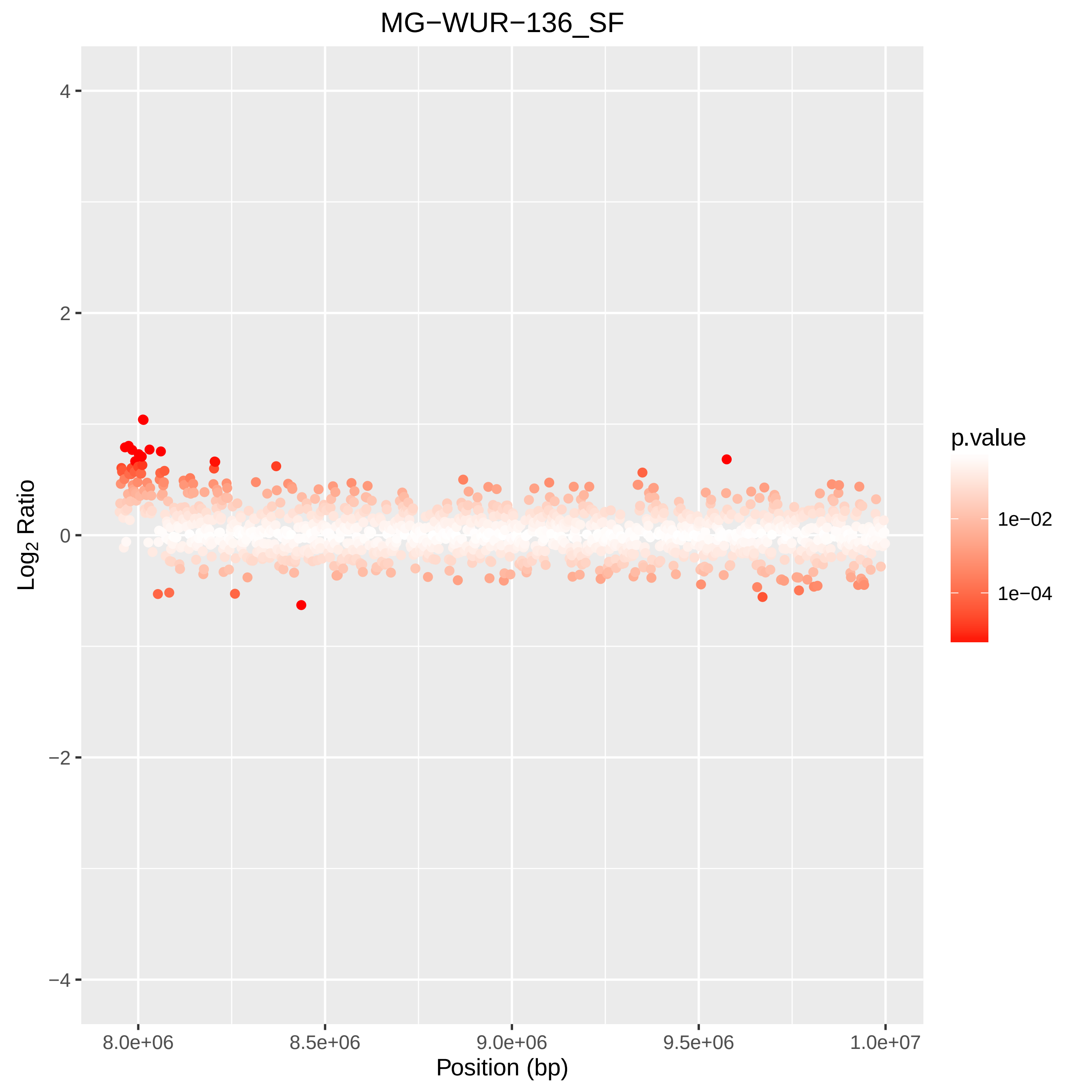
MG−WUR−136_SF
4
2
0
−2
−4
Log2 Ratio
8.0e+06
8.5e+06
9.0e+06
9.5e+06
1.0e+07
Position (bp)
p.value
1e−02
1e−04

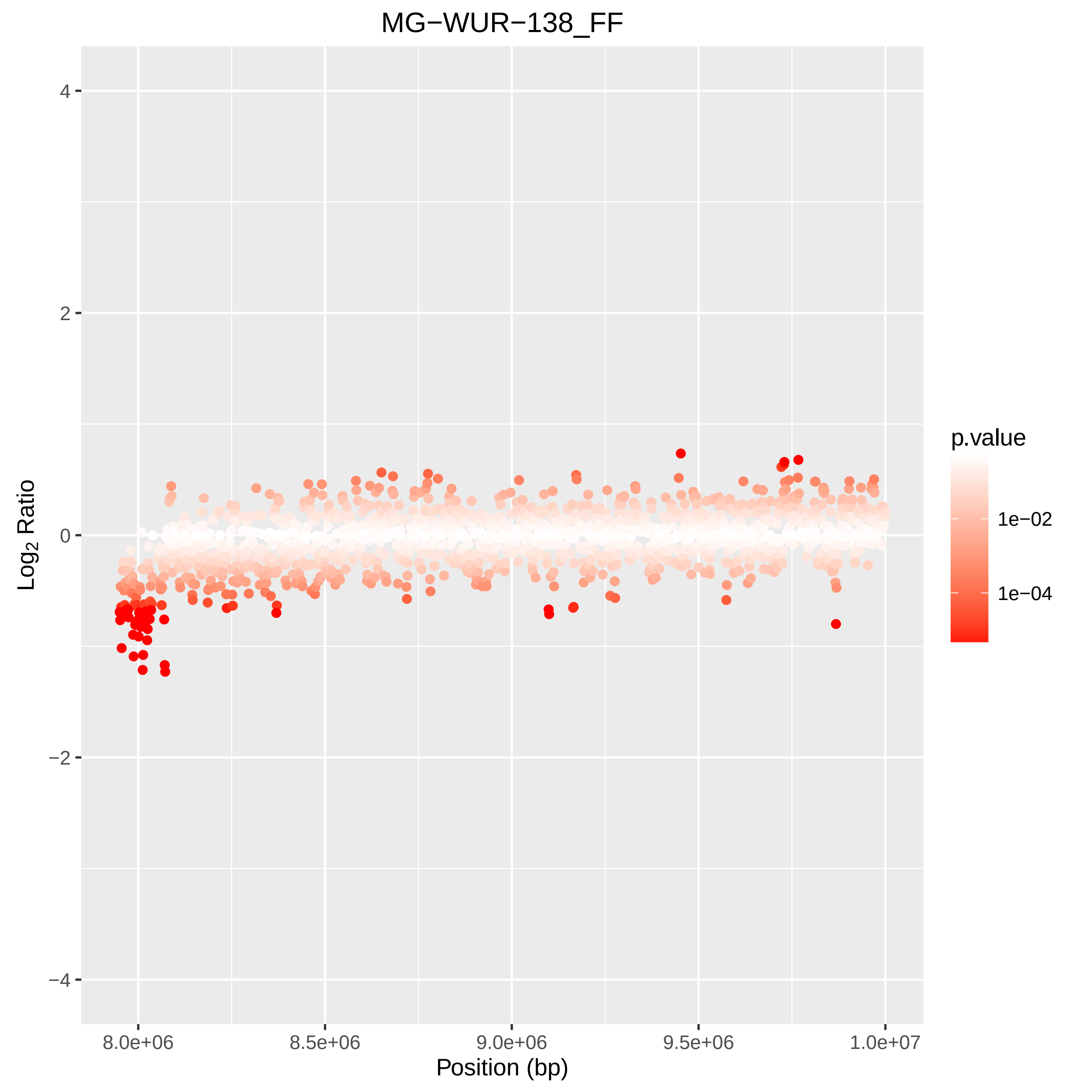

MG−WUR−138_FF
4
2
0
−2
−4
Log2 Ratio
8.0e+06
8.5e+06
9.0e+06
9.5e+06
1.0e+07
Position (bp)
p.value
1e−02
1e−04

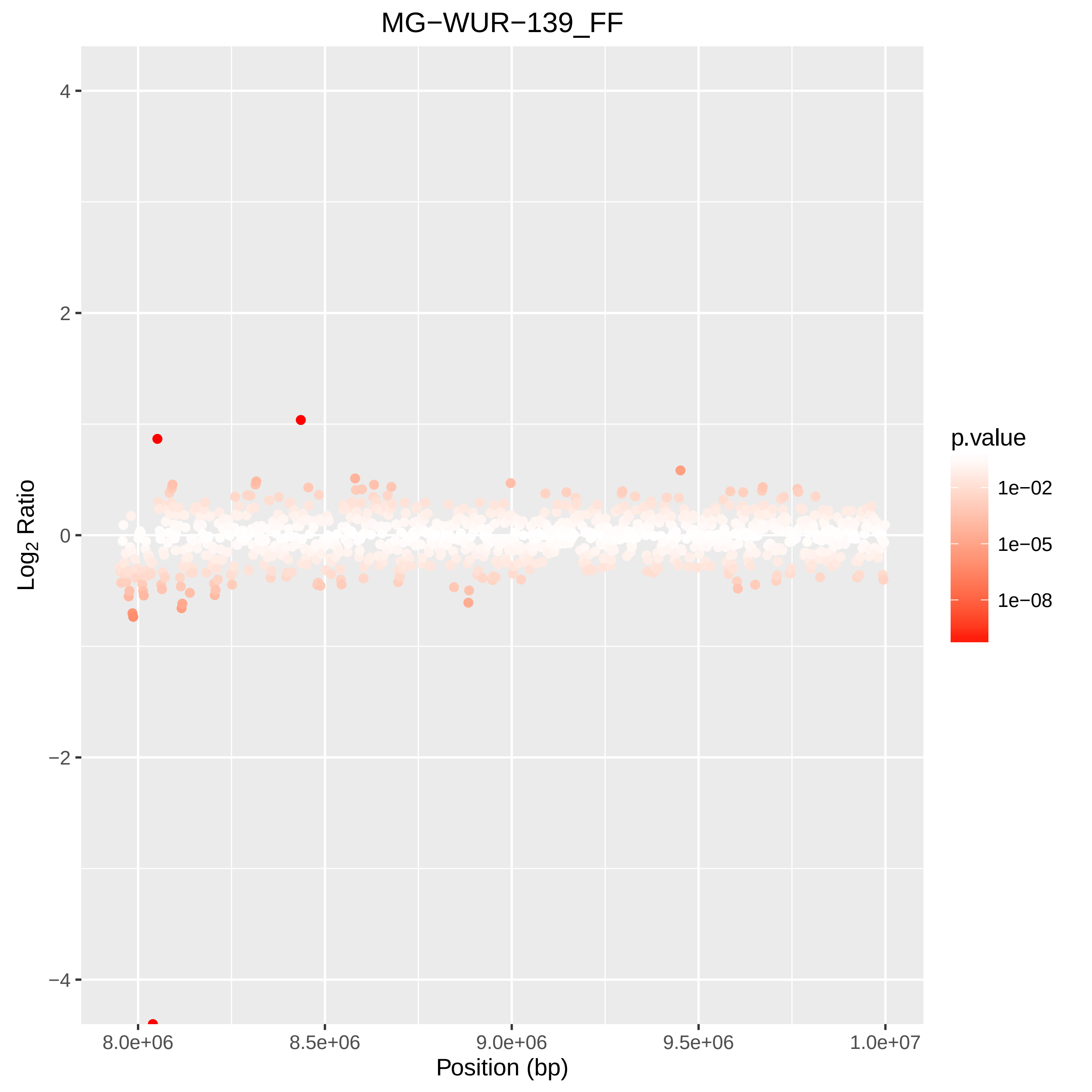

MG−WUR−139_FF
Log2 Ratio
4
2
0
−2
−4
8.0e+06
8.5e+06
9.0e+06
9.5e+06
1.0e+07
Position (bp)
p.value
1e−02
1e−05
1e−08

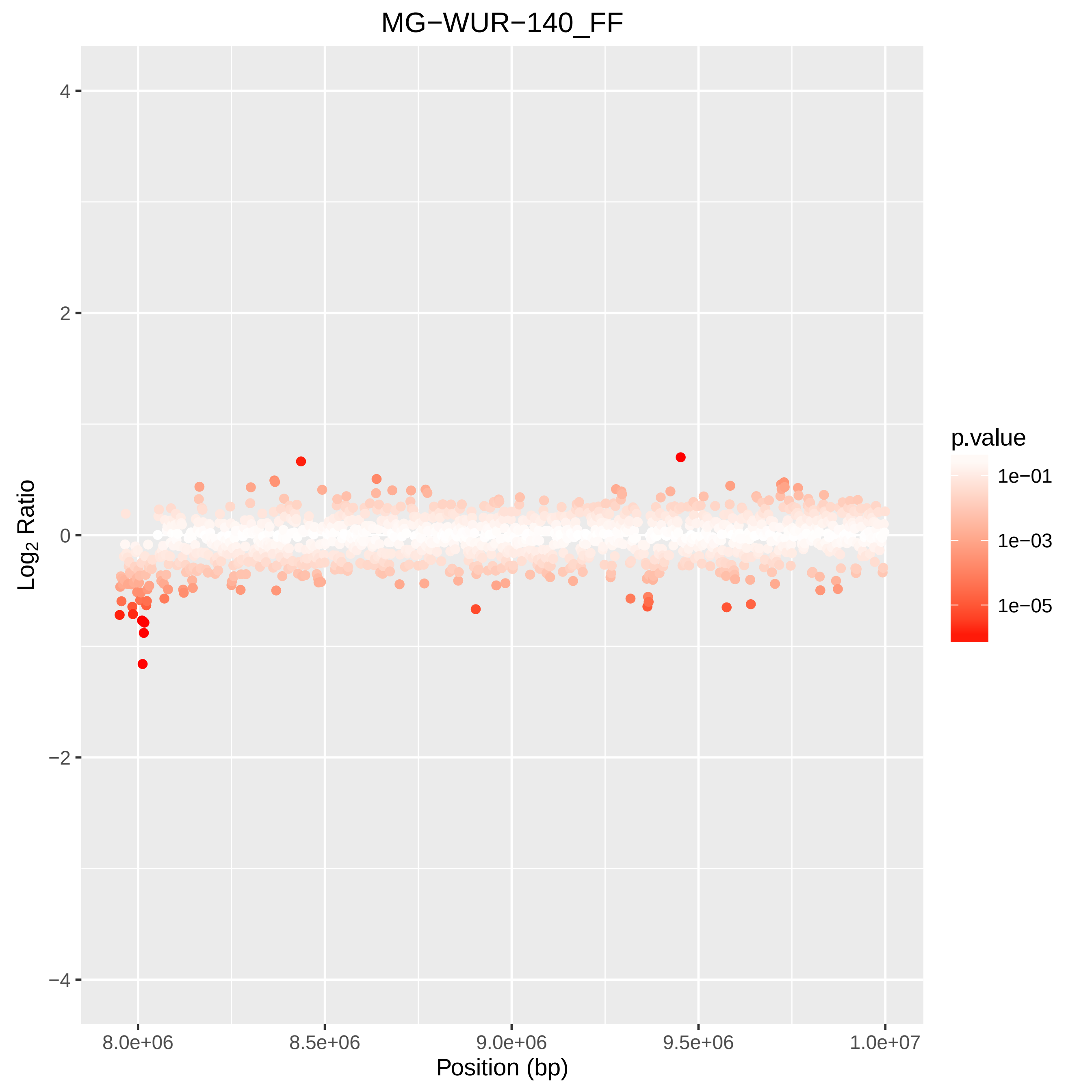
MG−WUR−140_FF
4
2
0
−2
−4
Log2 Ratio
8.0e+06
8.5e+06
9.0e+06
9.5e+06
1.0e+07
Position (bp)
p.value
1e−01
1e−03
1e−05

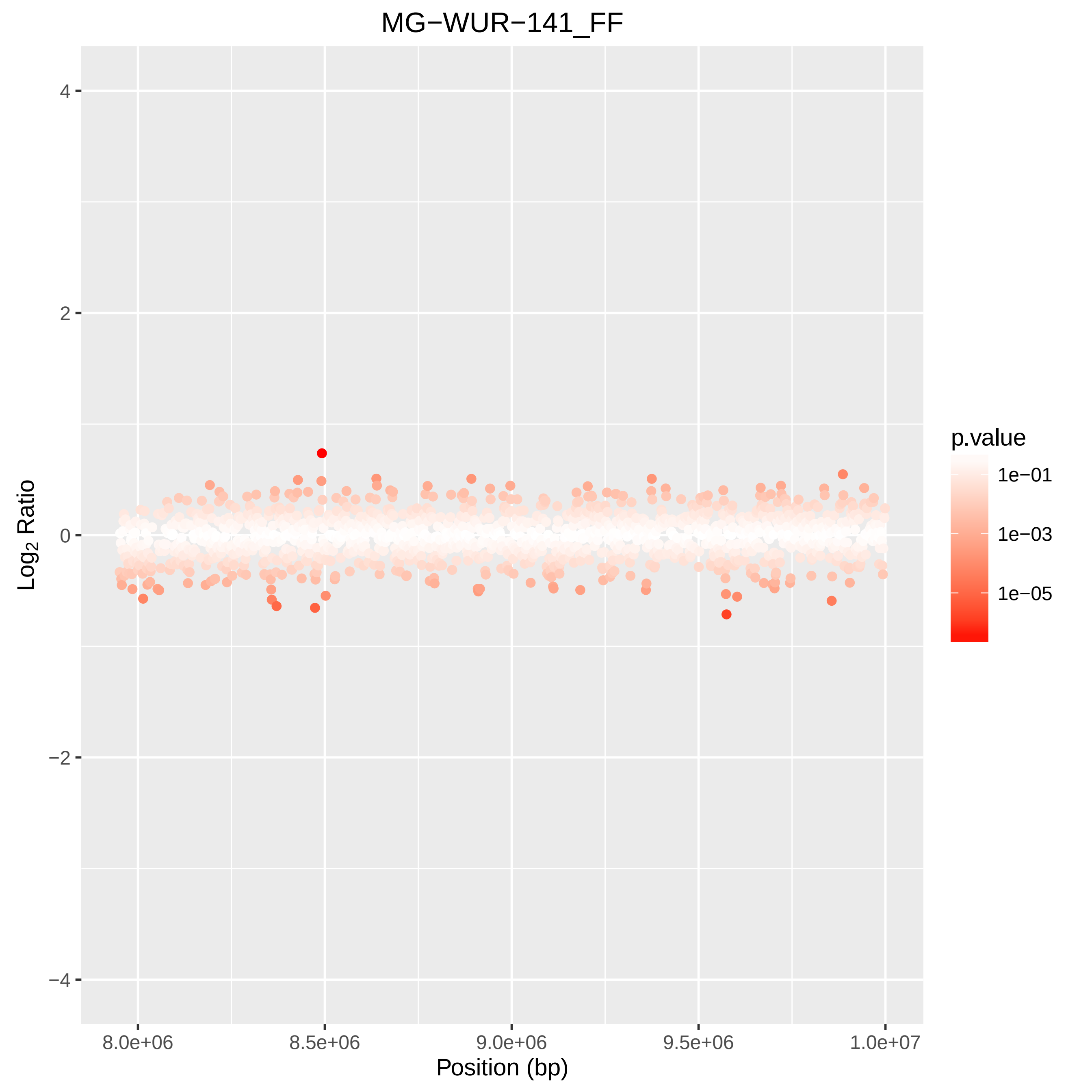

MG−WUR−141_FF
4
2
0
−2
−4
Log2 Ratio
8.0e+06
8.5e+06
9.0e+06
9.5e+06
1.0e+07
Position (bp)
p.value
1e−01
1e−03
1e−05

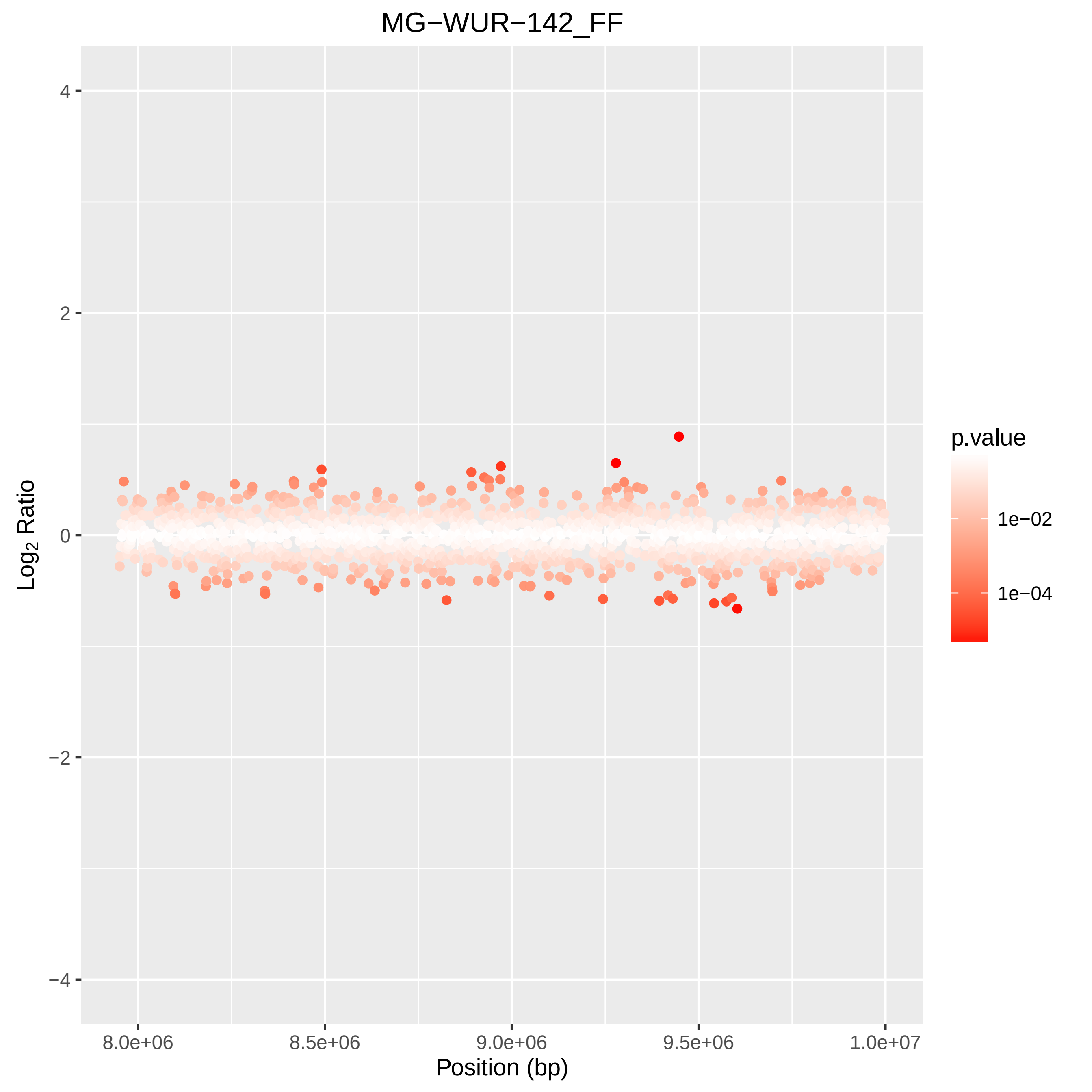

MG−WUR−142_FF
4
2
0
−2
−4
Log2 Ratio
8.0e+06
8.5e+06
9.0e+06
9.5e+06
1.0e+07
Position (bp)
p.value
1e−02
1e−04

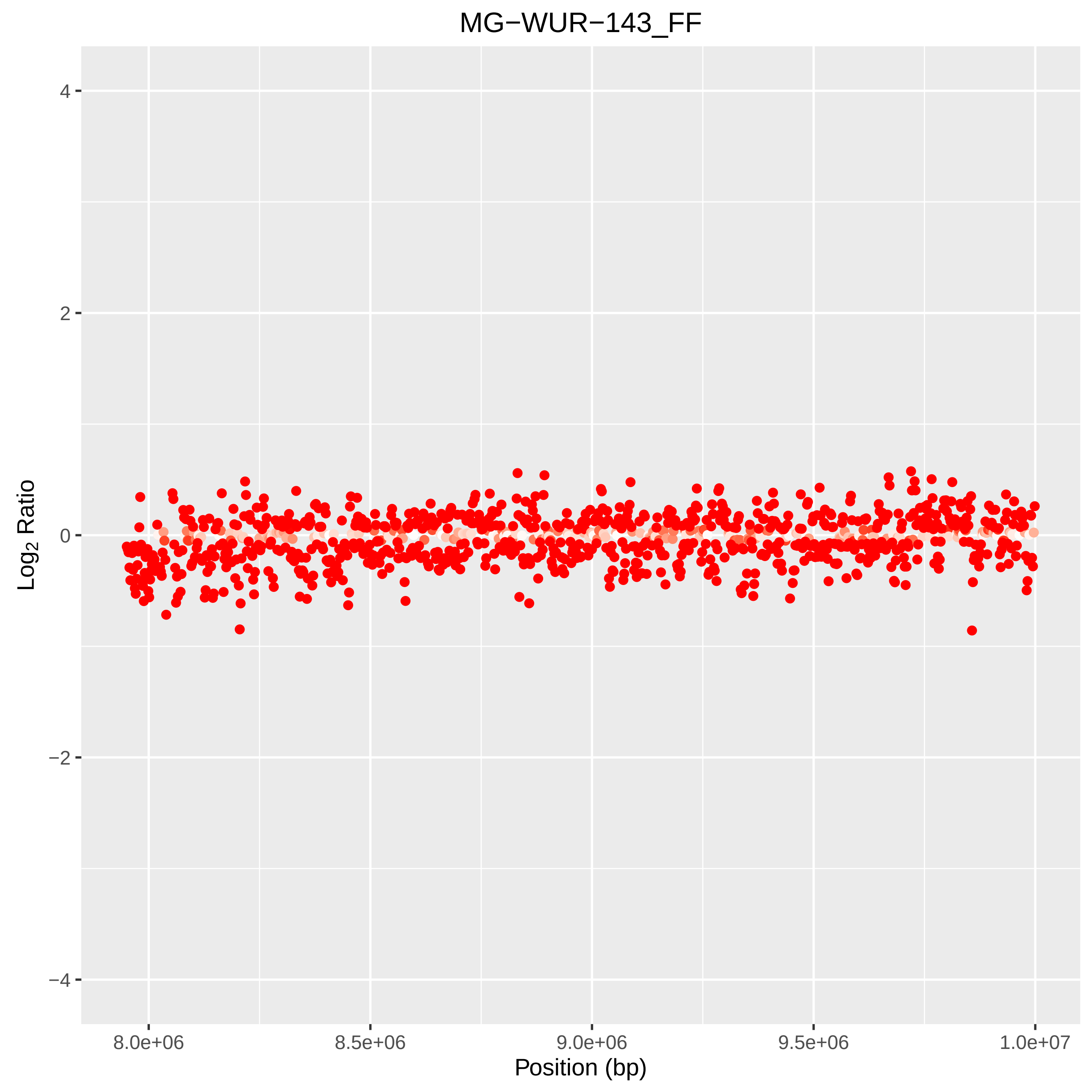
MG−WUR−143_FF
4
2
0
−2
−4
Log2 Ratio
8.0e+06
8.5e+06
9.0e+06
9.5e+06
1.0e+07
Position (bp)

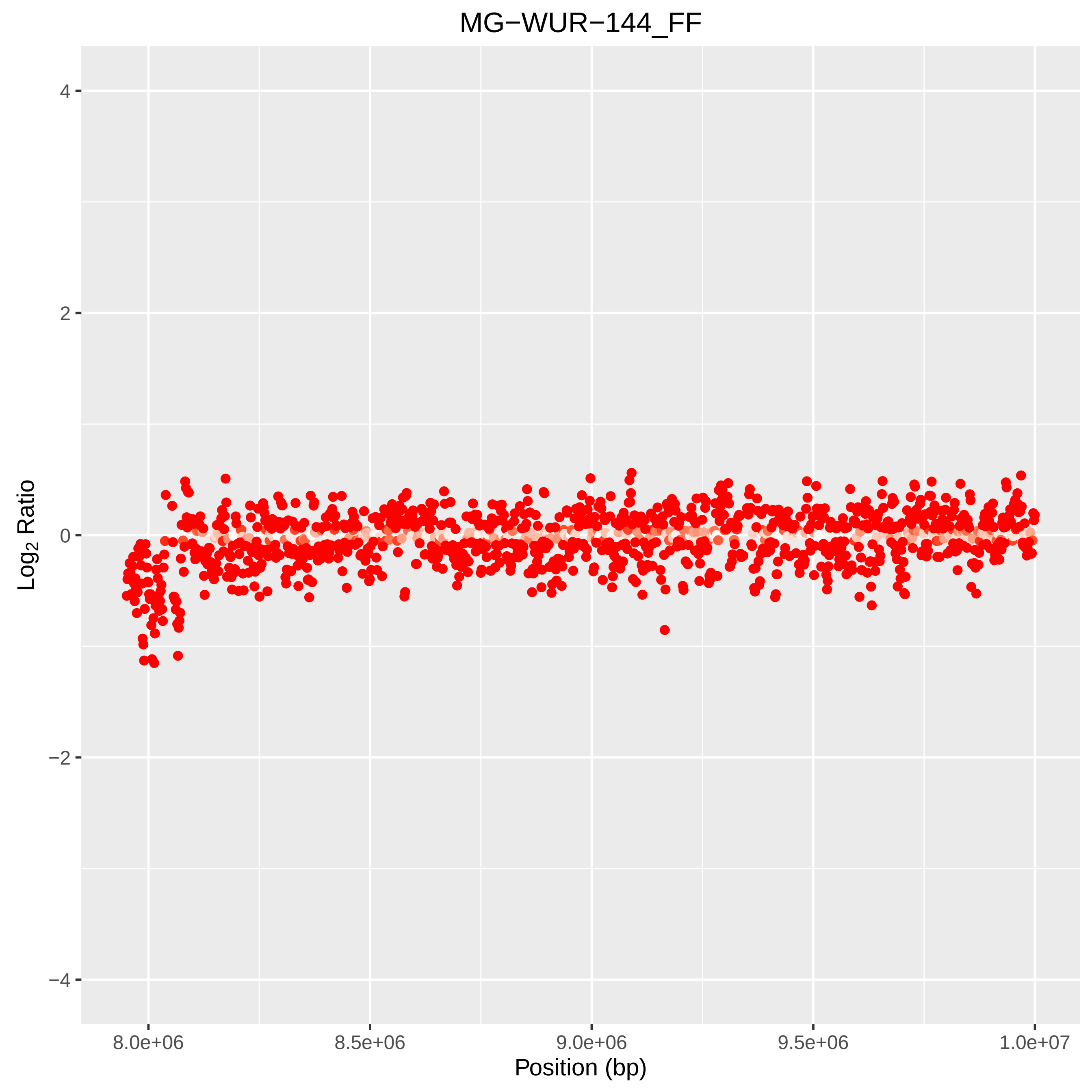

MG−WUR−144_FF
4
2
0
−2
−4
Log2 Ratio
8.0e+06
8.5e+06
9.0e+06
9.5e+06
1.0e+07
Position (bp)
